# Opposite Interactive Effects of Heat Wave and Cold Spell with Fine Particulate Matter on Pneumonia Mortality

**DOI:** 10.3390/toxics13080702

**Published:** 2025-08-21

**Authors:** Yi Zheng, Ruijun Xu, Yuling Chen, Yingxin Li, Yuxin Bi, Xiaohong Jia, Sirong Wang, Lu Luo, Jing Wei, Rui Wang, Chunxiang Shi, Ziquan Lv, Suli Huang, Gongbo Chen, Hong Sun, Bochao Sun, Nongping Feng, Yuewei Liu

**Affiliations:** 1 Department of Epidemiology, School of Public Health, Sun Yat-sen University, Guangzhou 510080, China; zhengy329@mail2.sysu.edu.cn (Y.Z.); xurj5@mail2.sysu.edu.cn (R.X.); gzcdc_chenyl@gz.gov.cn (Y.C.); liyx83@mail2.sysu.edu.cn (Y.L.); biyx5@mail2.sysu.edu.cn (Y.B.); jiaxh6@mail2.sysu.edu.cn (X.J.); wangsr23@mail2.sysu.edu.cn (S.W.); luolu23@mail2.sysu.edu.cn (L.L.); 2 Department of Evironmental Health, Guangzhou Center for Disease Control and Prevention, Guangzhou 510440, China; 3 Department of Atmospheric and Oceanic Science, Earth System Science Interdisciplinary Center, University of Maryland, College Park, MD 20740, USA; weijing_rs@163.com; 4 Luohu District Chronic Disease Hospital, Shenzhen 518020, China; wangrui.1983@hotmail.com; 5 Meteorological Data Laboratory, National Meteorological Information Center, Beijing 100081, China; shicx@cma.gov.cn; 6 Shenzhen Center for Disease Control and Prevention, Shenzhen 518055, China; lvziquan1984@126.com; 7 Department of Environment and Health, School of Public Health, Shenzhen University Medical School, Shenzhen University, Shenzhen 518055, China; huangsuli420@163.com; 8 Climate, Air Quality Research Unit, School of Public Health and Preventive Medicine, Monash University, Melbourne, VIC 3004, Australia; gongbo.chen1@monash.edu; 9 Department of Environment and Health, Jiangsu Provincial Center for Disease Control and Prevention, Nanjing 210009, China; hongsun@jscdc.cn; 10 Yancheng Center for Disease Control and Prevention, Yancheng 224002, China; sbc912034@163.com; 11 Department of Non-Communicable Disease Prevention and Control, Shenzhen Longgang Center for Chronic Disease Control, Shenzhen 518172, China

**Keywords:** extreme temperature events, PM_2.5_, pneumonia, synergistic effect, antagonistic effect

## Abstract

Exposure to extreme temperature events (ETEs) and ambient fine particulate matter (PM_2.5_) has been linked to an increased risk of pneumonia mortality, but their interactive effects remain largely unknown. We investigated 50,196 pneumonia deaths from 2015 to 2022 in Jiangsu province, China, with a time-stratified case-crossover design. An individual-level exposure to heat wave, cold spell, and PM_2.5_ was assessed at each subject’s residential address using validated grid datasets. Conditional logistic regression models integrated with a distributed lag nonlinear model were used to quantitatively estimate both independent and interactive effects. With different ETE definitions, the cumulative odds ratio (OR) of pneumonia mortality associated with heat wave and cold spell ranged from 1.22 (95% confidence interval [CI]: 1.14, 1.31) to 1.60 (1.40, 1.81), and from 1.08 (1.002, 1.17) to 1.18 (1.01, 1.38), respectively, while the OR for PM_2.5_ ranged from 1.013 (1.006, 1.021) to 1.016 (1.009, 1.024). We observed a synergistic effect (relative excess risk due to interaction [RERI] ranging from 0.40 [0.06, 0.76] to 1.16 [0.41, 2.09]) of co-exposure to heat wave and PM_2.5_, as well as an antagonistic effect (RERI ranging from −0.20 [−0.40, −0.03] to −1.02 [−1.78, −0.38]) of co-exposure to cold spell and PM_2.5_ on pneumonia mortality. It was estimated that up to 6.49% of pneumonia deaths were attributable to heat wave and PM_2.5_ exposures. We found that heat wave and cold spell interacted oppositely with PM_2.5_ to increase the odds of pneumonia mortality, highlighting the needs to reduce co-exposures to heat wave and PM_2.5_.

## 1. Introduction

As a severe acute respiratory disease, pneumonia continues to be a globally leading cause of morbidity and mortality [1]. According to the Global Burden of Diseases, Injuries, and Risk Factors Study (GBD), over 2.18 million lower respiratory infections (LRI, defined as pneumonia or bronchiolitis) deaths occurred in 2021, resulting in a mortality rate of 27.7 deaths per 100,000 people worldwide [2]. In China, the community-acquired pneumonia contributed to approximately 128,720 deaths and 111.98 years of life lost (YLLs) per 100,000 people in 2021 [3]. This situation becomes more severe due to a higher vulnerability of elderly individuals to pneumonia, posing a significant public health challenge [1,4]. It is indispensable to identify potential risk factors for pneumonia mortality to effectively formulate health policies and protect vulnerable populations.

As a matter of climate change, the adverse health effects caused by extreme temperature events (ETEs, including heat wave and cold spell) and ambient air pollution have drawn increasing concern worldwide [5,6,7]. Recent studies have identified ETEs and fine particulate matter (PM_2.5_) as potential risk factors for pneumonia death [8,9]. Given ETEs are projected to increase in frequency and intensity, and the opportunities to co-exposure to heat wave, cold spell, and PM_2.5_ are gradually increasing [10,11,12], it is of great importance and interest to recognize the pattern of interactive effects of these two risk factors on pneumonia mortality, which remains largely unknown. A recent time-stratified case-crossover study conducted in China during 2013–2019 reported that the risk of pneumonia death associated with PM_2.5_ exposure was higher in cold season compared with that in warm season [13], while another case-crossover study in Hong Kong, China identified significantly higher acute adverse effects of PM_2.5_ exposure on pneumonia mortality in warm climate [14]. Although these limited relevant studies provide certain evidence on potential interaction of ETEs and PM_2.5_ on pneumonia mortality, these findings remain inconclusive and clearly warrant further quantitative studies.

To fill this research gap, a population-based case-crossover study of 50,196 subjects was conducted to investigate both independent and interactive effects of exposure to heat wave and cold spell with PM_2.5_ on pneumonia mortality. Established exposure–response associations were further used to estimate excess mortality due to co-exposures to ETEs and PM_2.5_. In addition, stratified analyses were conducted by sex and age to detect potentially vulnerable populations.

## 2. Materials and Methods

### 2.1. Study Population

We obtained mortality data in Jiangsu province between 1 January 2015 and 30 November 2022 from the Jiangsu provincial mortality surveillance system. Using J12-J18 from the International Statistical Classification of Diseases and Related Health Problems, 10th revision (ICD-10), we identified 50,196 individuals dying from pneumonia as the underlying cause during the period. Specific information on date of birth, sex, residential address before death, and date of death was collected for each subject. The study protocol was approved by the Ethics Committee of School of Public Health, Sun Yat-sen University with a waiver of informed consent.

### 2.2. Study Design

This study utilized a time-stratified case-crossover design, which has been widely applied in evaluating the acute effects of both temperature and air pollution on a series of health outcomes [5,15,16,17]. This design was characterized that each subject served as its own control, making it possible to quantify the association between environmental risk factors and outcomes of interest by comparing the exposure levels on case day and control days. In this study, the case day was designated as the date of death for each subject, while the corresponding control days were identified as dates sharing the same month and day of week as the case day. For instance, if a case died from pneumonia on 13 October 2017 (Friday), its case day would be 13 October 2017, and all other Fridays in October 2017 (i.e., October 6, October 20, and October 27) would be selected as the comparable control days. Accordingly, the effects of time-invariant variables, long-term trends, and seasonality can be naturally controlled [18].

### 2.3. Exposure Assessment

Using the China Meteorological Administration Land Data Assimilation System (CLDAS version 2.0), we assessed daily heat wave and cold spell exposures [19,20,21]. We retrieved daily gridded mean temperature (°C) and relative humidity (%) data with a spatial resolution of 0.0625° × 0.0625° in Jiangsu province from 2015 to 2022, and used them to determine the heat index (HI) using the R package (version 4.4.1) *weathermetrics* [22]. HI can compressively reflect human-perceived temperature by combining temperature with humidity and has been emergingly used to explore the effect of meteorological factors on health [6,23]. To conduct sensitivity analyses, we used daily mean air temperature, specific humidity, wind speed, and surface pressure to calculate apparent temperature (AT; °C) [5,24]. We then employed the gridded data on HI to generate 12 daily gridded datasets for ETEs in Jiangsu province during 2015–2022. As suggested in prior studies, we identified a grid-specific heat wave as the HI equal to or exceeding a given threshold (i.e., the 90th, 92.5th, 95th, or 97.5th percentile of HI distribution during 2015–2022 in the grid [P90, P92.5, P95, or P97.5]) for at least 2, 3, or 4 days, while a cold spell was identified as HI equal to or below a threshold (P10, P7.5, P5, or P2.5) for at least 2, 3, or 4 days [25,26]. For each subject, we then extracted ETE exposures at her or his geocoded residential address on the case and control days.

Gridded data of PM_2.5_ (daily 24 h mean; 1 km × 1 km) and ozone (O_3_; daily maximum 8 h mean; 1 km × 1 km) in Jiangsu province during 2015–2022 were retrieved from our validated ChinaHighAirPollutants (CHAP) dataset (available at: https://weijing-rs.github.io/product.html; accessed on 1 January 2024). CHAP is a high-quality air pollution dataset that was generated through satellite remote sensing and machine learning [27,28]. The cross-validated coefficients of determination (R^2^) for PM_2.5_ and O_3_ were 0.92 and 0.92, while the root-mean-square deviations (RMSE) were 10.8 and 13.5 µg/m^3^, respectively [27,28]. Similarly to ETEs, PM_2.5_ and O_3_ exposure was assessed by extracting their concentrations based on each subject’s residential address.

### 2.4. Statistical Analysis

A conditional logistic regression model with a distributed lag nonlinear model (DLNM) was used to investigate the nonlinear and linear association of ETE and PM_2.5_ exposure with pneumonia mortality. To explore potential lag effects, we set a maximal lag period of 6 days by building a cross-basis function of exposure based on prior knowledge that the short-term effects of PM_2.5_ commonly last for about one week [29]. We used a cross-basis function to generate the basis metrics for the two dimensions of both exposure and lags. The exposure–response function for ETEs was built using a “strata” function (0, neither heat wave nor cold spell; 1, heat wave; 2, cold spell); the lag-response function was constructed using a natural cubic spline with 2 knots placed at equal interval in the logarithmic scale [29]. For PM_2.5_, the exposure–response and lag-response associations were initially built with a natural cubic spline with 3 degrees of freedom (*df*) and 2 interval knots at equally spaced point in the logarithmic scale, respectively. A likelihood ratio test was then applied to test potential nonlinear association between PM_2.5_ exposure and pneumonia mortality. If the association did not depart from linearity, we would use the DLM using a “lin” function for exposure–response associations and polynomial function with 3 *df* for lag-response associations for PM_2.5_ exposure [30]. Odds ratio (OR) and its 95% confidence interval (CI) were calculated to assess the cumulative effects of ETEs and PM_2.5_.

Based on the median exposure to PM_2.5_ (cut-off values: 40.8 μg/m^3^), we divided PM_2.5_ exposure into high-level and low-level, and constructed a categorical variable to reflect different exposure scenarios including: (1) neither heat wave nor cold spell (non ETEs) and low-level PM_2.5_ (reference level); (2) non ETEs and high-level PM_2.5_; (3) heat wave and low-level PM_2.5_; (4) cold spell and low-level PM_2.5_; (5) heat wave and high-level PM_2.5_; and (6) cold spell and high-level PM_2.5_. In building the cross-basis function in DLNMs, the “strata” function was used for this categorical exposure. The estimated ORs and their 95% CIs were then used to calculate the relative excess risk due to interaction (RERI, indicating the direction of additive interaction), attributable proportion due to interaction (AP, indicating the proportion of the risk associated with interaction), and synergy index (S, indicating whether the risk associated with co-exposure is greater than risk associated with single-exposure), which have been extensively used in quantitatively assessing additive interactions [31,32]. We applied a bootstrap method to estimate the 95% CI using 1000 replicate samples [33].

(1) $$RERI={OR}_{11}-{OR}_{10}-{OR}_{01}+1$$

(2) $$AP=\frac{RERI}{{OR}_{11}}$$

(3) $$S=\frac{{OR}_{11}-1}{\left({OR}_{10}-1)+({OR}_{01}-1\right)}$$

where OR_11_ refers to the estimated OR for scenario 5 or 6; OR_10_ refers to the OR for scenario 3 or 4; OR_01_ refers to the OR for scenario 2. An RERI of 0, AP of 0, and S of 1 indicate no interaction or exact additivity. An RERI and AP greater than 0, and S greater than 1 indicate a positive interaction (synergistic effect), while a negative RERI and AP, and S smaller than 1 indicate a negative interaction (antagonistic effect) [32].

To estimate excess pneumonia mortality, we calculated the excess fraction and number of excess deaths due to heat wave, cold spell, and high-level PM_2.5_ based on the estimated associations and their exposures [34]. We further calculate 95% empirical confidence intervals (eCIs) using Monte Carlo simulations [34].

(4) $${Excess\;fraction}_{x,t}=1-e^{\left(-\sum_{l=l_{0}}^{L}\beta_{x_{t-l},l}\right)}$$

(5) $${Number\;of\;excess\;death}_{x,t}={Excess\;fraction}_{x,t}\times N_{t}$$

where *x* refers to heat wave, cold spell, and/or high-level PM_2.5_ exposure; *t* refers to the date of the lag period; *l* refers to lag days; *l*_0_ refers to minimum lags; *L* refers to maximum lags; *β* refers to the coefficient associated with exposure to *x*; N*_t_* refers to the number of deaths at time *t*.

To identify the effects on different populations, we performed stratified analyses by age (≤80 years old, >80 years old) and sex (women, men). A 2-sample *z*-test was used to compare the significant difference between different stratum-specific estimates [35].

(6) $$z=\frac{\beta_{1}-\beta_{2}}{\sqrt{{{SE}_{1}}^{2}+{{SE}_{2}}^{2}}}$$

where *β*_1_ and *β*_2_ refer to coefficients of the conditional logistic regression models, respectively; *SE*_1_ and *SE*_2_ refer to the corresponding standard errors.

We conducted the following sensitivity analyses to examine the robustness of our findings. To account for potential confounding by other pollutants, we fitted 2-pollutant models by further including O_3_ (a typical gaseous pollutant) by constructing a similar cross-basis function as PM_2.5_ in the model. In addition, like most studies, we conducted analyses in different seasons (warm: May to October; cold: November to March) to indirectly identify potential interactions. We also used the interim target 3 value (IT3: 37.5 μg/m^3^) of PM_2.5_ in World Health Organization (WHO) air quality guidelines (AQGs) to categorize PM_2.5_ exposure in the interaction analysis. Finally, we employed air temperature and AT instead of the HI to define heat wave and cold spell, and explore interactive effects with PM_2.5_. Similarly to HI, AT is a commonly used indicator of perceived temperature, but it incorporates humidity and wind speed in addition to air temperature. The use of air temperature and AT enabled us to assess the robustness of our findings across different temperature metrics. All analyses were analyzed using R version 4.4.1. A 2-sided *p* < 0.05 was defined as statistically significant.

## 3. Results

### 3.1. Descriptive Statistics

**Figure 1:**
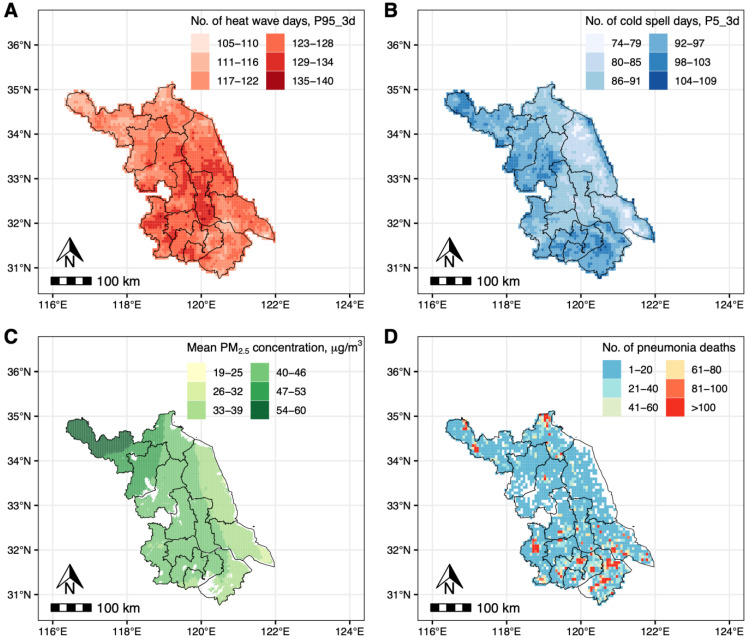
Spatial distribution of heat wave (P95_3d), cold spell (P5_3d), mean PM_2.5_ concentration, and pneumonia mortality in Jiangsu province, China, during 2015–2022. Spatial distribution of number of heat wave days of P95_3d (**A**), number of cold spell days of P5_3d (**B**), mean PM_2.5_ concentration (**C**), and study subjects (**D**). The spatial resolution is 0.0625° × 0.0625° (**A**), 0.0625° × 0.0625° (**B**), 1 km × 1 km (**C**), and 0.0625° × 0.0625° (**D**). PM_2.5_—fine particulate matter.

During the study period, we identified 50,196 deaths from pneumonia, yielding 50,196 case days and 169,721 control days. Table 1 gives the basic characteristics of the subjects. Men accounted for 52.3% of the subjects. About 71.5% of the subjects died after 80 years old. The mean concentration of PM_2.5_, O_3_, and HI on the date of death was 49.0 μg/m^3^, 103.1 μg/m^3^, and 15.7 °C, respectively (Table 2). The threshold of heat wave (90th, 92.5th, 95th, or 97.5th percentile of HI) was 31.6, 33.6, 35.8, and 38.7°C, and threshold of cold spell (2.5th, 5th, 7.5th, or 10th percentile of HI) was 0.4, 1.7, 2.5, and 3.2°C, respectively (Table S1). The spatial distribution of number of heat wave days (A), number of cold spell days (B), mean PM_2.5_ concentration (C), and number of pneumonia deaths (D) is demonstrated in Figure 1. Table S2 shows the number of pneumonia deaths in different exposure conditions. Overall, more subjects died on cold spell days, during which a higher proportion of days were accompanied with high-level PM_2.5_ compared with that on heat wave days.

### 3.2. Exposure–Response Analysis

As presented in Table 3, the risk for pneumonia mortality positively increased following a single exposure to heat wave, cold spell, and PM_2.5_ conditions (all *p* < 0.05), though the association for cold spell in P5_3d was statistically insignificant. In general, the effects of short-term exposure to heat wave were stronger than that of cold spell, with the OR of heat wave ranging from 1.22 (P90_3d) to 1.60 (P97.5_3d) and 1.08 (P5_2d) to 1.18 (P2.5_4d) for cold spell, respectively; in addition, the associations were gradually stronger with stricter HI threshold. After adjusting for ETEs with different definitions, the odds of pneumonia mortality associated with PM_2.5_ exposure were significant and stable, and the associations did not depart from linearity (Figure S1).

Figure S2 illustrates lag structures of the significant association between heat wave and cold spell and pneumonia mortality. Under most heat wave definitions (i.e., P95_3d), the odds of heat wave peaked on the lag 0 day and then attenuated to null by lag 1 day but slightly increased again at lag 3–4 days. In contrast, under most definitions (i.e., P5_3d), cold spell showed certain delayed effects, with the odds beginning and peaking at lag 1 day or lag 2 day, and then attenuating. The lag patterns of PM_2.5_ showed a similar trend to heat wave, with the strongest association at lag 0 day, decreased at lag 1 day, and attenuated to null afterward (Figure S3).

### 3.3. Interactive Effects

**Figure 2:**
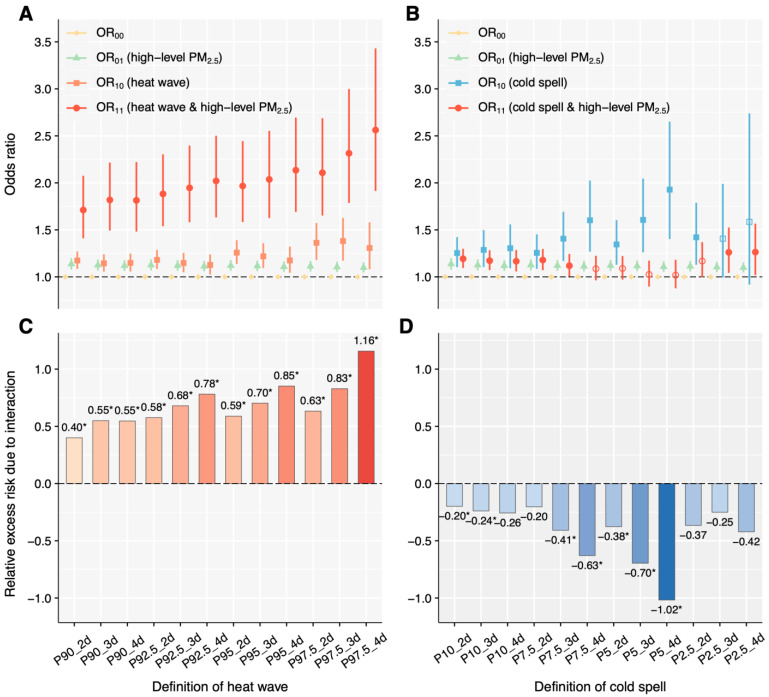
Cumulative association and additive interactive effects of exposure to ETEs and high-level PM_2.5_ on pneumonia mortality. Cumulative association of exposure to heat wave and high-level PM_2.5_ (**A**), cumulative association of exposure to cold spell and high-level PM_2.5_ (**B**), interactive effects of exposure to heat wave and high-level PM_2.5_ (**C**), and interactive effects of exposure to cold spell and high-level PM_2.5_ (**D**). Solid markers and “*” indicate statistical significance, while hollow markers represent non-significance. RERI > 0 indicates a positive interaction (synergistic effect), while RERI < 0 indicates a negative interaction (antagonistic effect). OR, odds ratio; PM_2.5_, fine particulate matter; RERI: relative excess odds due to interaction; ETEs: extreme temperature events.

Figure 2, as well as Tables S3 and S4, illustrates the positive interactive effects of heat wave and high-level PM_2.5_ on pneumonia mortality. The OR of heat wave and high-level PM_2.5_ co-exposure over lag 0–6 day (OR_11_) was much higher than that of heat wave only (OR_10_) and high-level PM_2.5_ only (OR_10_) across all definitions of heat wave, with interactive effects that were all significantly synergistic (RERI > 0, AP > 0, and S > 1). The RERI, AP, S were ranged from 0.40 to 1.16, 0.23 to 0.45, and 2.29 to 4.25, respectively. In addition, the RERI, AP, and S appeared to be higher with increasing severity of heat wave (i.e., higher HI threshold, or more consecutive days). In contrast, the OR_11_ of exposure to both cold spell and high-level PM_2.5_ was generally lower than OR_10_ and/or OR_01_, and the RERI was lower than 0 (ranging from −0.20 to −1.02), indicating an antagonistic effect between cold spell and high-level PM_2.5_ on pneumonia mortality (Figure 2, Tables S3 and S4).

**Figure 3:**
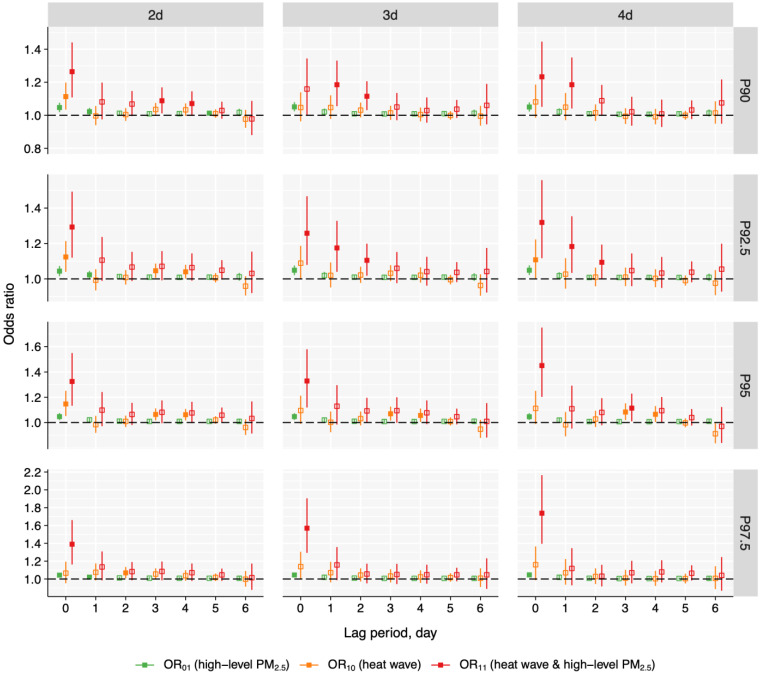
Overall lag structure for the association of PM_2.5_, heat wave, and co-exposure with pneumonia mortality in Jiangsu province, China during 2015–2022. Solid markers indicate statistical significance, while hollow markers represent non-significance. PM_2.5_, fine particulate matter.

**Figure 4:**
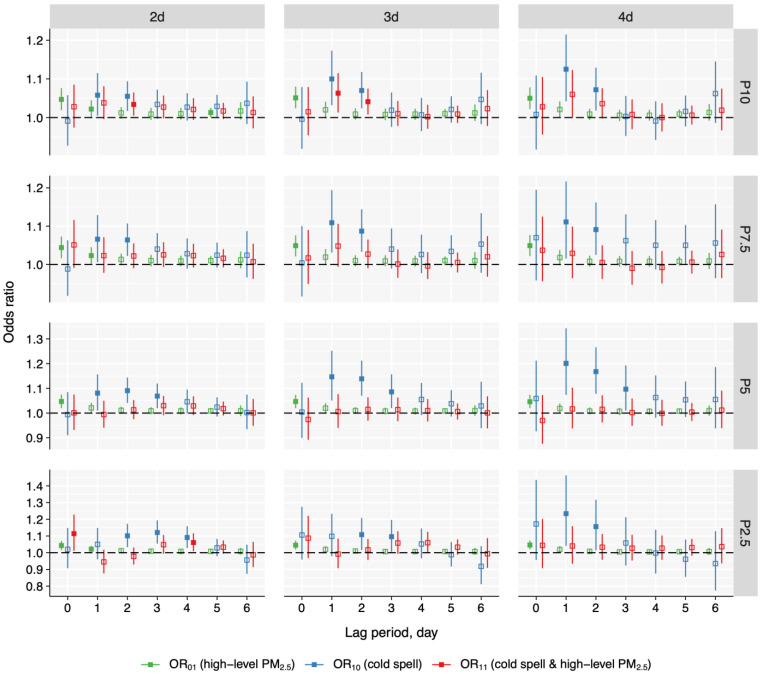
Overall lag structure for the association of PM_2.5_, cold spell, and co-exposure with pneumonia mortality in Jiangsu province, China during 2015–2022. Solid markers indicate statistical significance, while hollow markers represent non-significance. PM_2.5_, fine particulate matter.

The overall lag structures of heat wave, cold spell, and high-level PM_2.5_ are shown in Figure 3 and Figure 4. Except for the P90_3d and P97.5_2d definitions, the lag patterns for exposure to heat wave and high-level PM_2.5_), heat wave only, and high-level PM_2.5_ only all peaked at lag 0 day and then attenuated. For certain definitions (i.e., P90_2d and P95_3d), the exposure to heat wave and high-level PM_2.5_ slightly increased on lag 3 day or lag 4 day. In comparison, under most definitions (i.e., P7.5_2d, P5_2d, and P2.5_2d), co-exposure to cold spell and high-level PM_2.5_ showed different lag structures to exposure to cold spell and high-level PM_2.5_ and cold spell. For instance, for P5_2d, the odds of cold spell and high-level PM_2.5_ exposure showed a downward trend at lag 0 and 1 day, and slightly escalated at lag 2 and 3 day, then continuously decreased till lag 6 day. Conversely, the OR_10_ of cold spell exposure increased and peaked at lag day 2 and then attenuated, while the odds of exposure to PM_2.5_ peaked at a concurrent day and then decreased.

**Figure 5:**
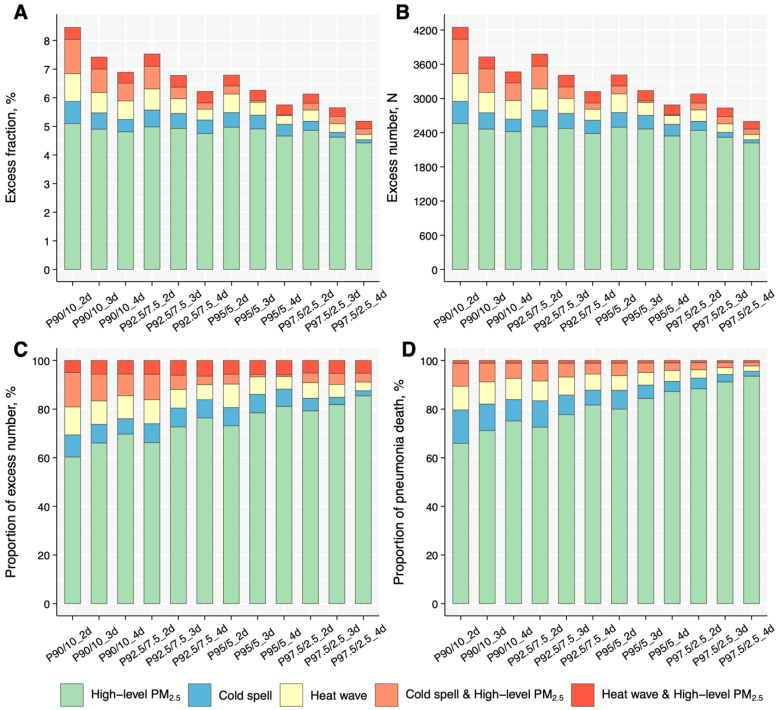
Excess fraction and number of excess deaths from pneumonia due to exposure to ETEs and high-level PM_2.5_. Excess fraction of pneumonia due to exposure to heat wave, cold spell, and high-level PM_2.5_ (**A**), number of excess deaths of pneumonia due to exposure to heat wave, cold spell, and high-level PM_2.5_ (**B**), percent of excess deaths of pneumonia due to exposure to heat wave, cold spell, and high-level PM_2.5_ (**C**), and percent of pneumonia deaths in different exposure (**D**). ETEs, extreme temperature events; PM_2.5_, fine particulate matter.

As shown in Figure 5 and Table S5, we estimated that 4.91% (95% CI: 2.69%, 7.00%), 0.45% (95% CI: 0.24%, 0.67%), and 0.36% (95% CI: 0.26%, 0.45%) of the pneumonia deaths were attributable to exposure to PM_2.5_, heat wave, and their co-exposures under a definition of P95_3d, corresponding to 2462 (95% CI: 1396, 3552), 227 (95% CI: 108, 338), and 180 (95% CI: 127, 222) deaths, respectively. For cold spell of P5_3d, the excess fraction of deaths for cold spell and its co-exposure to PM_2.5_ was 0.48% (95% CI: 0.22%, 0.70%) and 0.06% (95% CI: −0.27%, 0.37%), respectively (Figure 5, Table S5).

### 3.4. Stratified Analysis

Except for the stronger adverse effect of cold spell under P2.5_4d among individuals ≤ 80 years, the results of the stratified analysis did not show statistically different association of ETEs or PM_2.5_ with pneumonia mortality by sex or age (Tables S6, S7, S9 and S10). Overall, the synergistic effect of heat wave and high-level PM_2.5_ and antagonistic effect of cold spell and high-level PM_2.5_ remained among men, but not among women (Table S8). Compared with individuals > 80 years, individuals ≤ 80 years exhibited generally stronger synergistic effects of heat wave and high-level PM_2.5_. Under most cold spell definitions (except P10_4d), cold spell and high-level PM_2.5_ only demonstrated antagonistic effects in individuals ≤ 80 years (Table S11).

### 3.5. Sensitivity Analysis

By further adjusting for O_3_ in the model, we observed similar associations and effects of exposure to ETEs, and PM_2.5_ with pneumonia mortality (Tables S12 and S13). Compared with cold season, we observed significantly stronger adverse effects of PM_2.5_ exposure on pneumonia in warm season (Table S14). In addition, categorizing the PM_2.5_ exposure using 37.5 μg/m^3^ (Tables S15–S17) and using air temperature and apparent temperature instead of the HI to define heat wave and cold spell (Tables S18–S21) also yielded similar results.

## 4. Discussion

This is the first study to systematically explore the interactive effects of short-term exposure to ETEs with PM_2.5_ in relation to pneumonia mortality. In this population-based case-crossover study with an individual-level exposure assessment, we identified robust associations of heat wave, cold spell, and PM_2.5_ exposure with an increased odds of pneumonia mortality, and found a synergistic effect between heat wave and PM_2.5_ but an antagonistic effect between cold spell and PM_2.5_. In general, these interactions appeared to be stronger among men and individuals ≤ 80 years.

To date, only a few studies in Australia, Japan, Thailand, and China have investigated the adverse effects of heat wave or cold spell exposure on pneumonia mortality [36,37,38,39,40]. Despite differences in study design, exposure assessment, and the definition of ETEs, the findings of these studies were generally consistent with our study that both heat wave and cold spell exposure were significantly associated with pneumonia mortality. In contrast, the association between PM_2.5_ exposure and pneumonia mortality has been extensively studied, and the findings were overall conclusive and consistent with our study [41,42,43].

To our knowledge, no studies have quantitatively explored the interaction of co-exposure to heat wave, cold spell, and PM_2.5_ on pneumonia mortality. Limited studies have indirectly investigated the interaction by conducting stratified analyses by season (warm, cold) or levels of PM_2.5_ exposure, and the findings are mixed. A case-crossover study in Hong Kong, China reported a higher risk of pneumonia death due to PM_2.5_ exposure in warm climate (6.17%) than that in cool climate (−4.06%) [14]. Conversely, a nationwide case-crossover study in China demonstrated a stronger association in cold season (2.71% for viral pneumonia and 2.33% for bacterial pneumonia), though the effect modification by season did not reach statistical significance [13]. Another ecological study across 482 cities globally in 2000–2018 identified higher adverse effects of heat at high PM_2.5_ exposures (14.32%) than that at low PM_2.5_ exposures (7.65%) [44]. Consistent with our results, this limited evidence suggests synergistic adverse effects of co-exposure heat wave and high-level PM_2.5_. The novel findings from our study reveal antagonistic effect between cold spell and PM_2.5_ insights that heat wave and cold spell can interact differently with PM_2.5_ to induce pneumonia mortality. This antagonistic effect was also identified for mortality from respiratory diseases (including pneumonia) in an ecological study in Shanghai, China, under several definitions for cold spell [45], although a synergy was observed under some other definitions and in another time-series study in Xining, China [46].

The opposite interactive effects on the respiratory system can be explained by some biological mechanisms. PM_2.5_ can induce neutrophilic pulmonary inflammation, generate oxidative stress, and therefore result in the development or aggravation of pneumonia [47]. During heat waves, extremely high temperatures can significantly increase the respiratory rate of human, which leads to more inhalations of PM_2.5_ and exacerbate respiratory damage to pneumonia patients [48]. Moreover, exposure to heat wave would impair virus-specific adaptive immunity, thereby synergistically making the lung more susceptible to virus’ infection carried by PM_2.5_ [49,50]. During cold spell, compared with extreme heat that only appear in specific days, winter with shorter days and adverse weather conditions (including cold wind and high precipitation) would last longer; therefore, individuals tend to significantly reduce their outdoor activities in winter, and this kind of more indoor activities would protect themselves from co-exposures to cold spell and high-level PM_2.5_ [51]. This behavioral adaptation is reinforced by the higher electricity consumption in winter. A smart meter data from 2970 households in Jiangsu indicated that 87.3% of residents had winter electricity consumption peaks equal to or higher than summer peaks, suggesting a higher reliance on electric or other household heating devices in winter compared to air conditioning and other cooling devices in summer [52]. Additionally, low temperature can decrease the physiological responses, reduce the respiratory overall responsiveness for PM_2.5_ exposure, and potentially diminish the harmful effects of PM_2.5_ [53].

We provide novel evidence that men and individuals ≤ 80 years were more vulnerable to the interaction of ETEs and high-level PM_2.5_. Several mechanisms may relate to the vulnerability of synergistic effects and antagonistic effects on pneumonia mortality. Compared with women, men are overrepresented in outdoor professions and tend to experience higher activity levels, leading to more opportunities for heat wave and PM_2.5_ co-exposure [54]. During cold spell, men can mitigate the impact of cold spell with higher blood perfusion, vasoconstriction, and heat conservation, reducing vulnerability to pneumonia mortality when exposure to cold spell and PM_2.5_ [55]. Individuals ≤ 80 years may synergistically increase the vulnerability to both heat wave and PM_2.5_ through greater physiological responsiveness (i.e., metabolic rate) [56]. Conversely, the antagonistic effects observed during cold spells among individuals ≤ 80 years could be attributed to the greater thermoregulatory responses to cold exposure [57]. This nuanced difference in outdoor activities and thermoregulatory responses among different sex and age highlights the complex interaction between heat wave, cold spell, and high-level PM_2.5_. Our findings highlight critical needs to encourage the government, health sectors, and individuals to collaborate to implement effective prevention and protective measures based on individual and environmental conditions.

Pneumonia remains a significant public health concern, with considerable mortality and healthcare burden globally [1]. Our study provides compelling evidence that heat wave and PM_2.5_ interact synergistically to increase the risk of pneumonia mortality, emphasizing the urgency for policymakers and individuals with pneumonia to implement strategies to minimize exposure to both heat wave and PM_2.5_. In contrast, we found an antagonistic interaction between cold spell and PM_2.5_, which may not necessarily mean that cold spell can protect human against PM_2.5_. Instead, considering the substantial disease burden of PM_2.5_ exposure, adaptive behaviors such as reducing outdoor activities during cold spell should be emphasized to help mitigate PM_2.5_ exposures. Overall, policymakers need to recognize the complex interaction of heat wave, cold spell, and PM_2.5_, and develop targeted strategies to reduce pneumonia deaths from the rising threat of climate change and the increasing disease burden of PM_2.5_ [7].

One major strength of our study is that we used HI as the temperature metric to define and assess heat wave and cold spell, as it can better capture an individual’s perceptions of temperature by accounting for humidity and reflecting health effects more accurately [58]. In addition, we assessed the heat wave, cold spell, and PM_2.5_ exposure using validated datasets with high spatiotemporal resolutions, which can help obtain more accurate estimates. Yet this study also has certain limitations. First, like most prior studies, we did not measure individual exposure directly, therefore we were unable to account for time-activity patterns (e.g., the allocation of each subject’s time at work, home, and school) and personal behaviors (e.g., using the air conditioner to reduce heat wave exposure or staying indoors to reduce cold spell exposure). Exposure misclassifications of ETE, PM_2.5_, and O_3_ were inevitable, though these misclassifications were more likely to be non-differential and bias our results towards the null [59]. Second, it is essential to acknowledge the possibility of unmeasured confounders (i.e., smoking and drinking), which may lead to bias in our estimates. Third, one should exercise caution when extrapolating our findings to diverse populations, because the subjects were exclusively from one province in China, which has a temperate climate. The magnitude of the independent and interactive effects of ETEs and PM_2.5_ may differ in regions with warmer or colder climates, potentially affecting generalizability.

## 5. Conclusions

We found robust associations of exposure to heat wave, cold spell, and PM_2.5_ with pneumonia mortality, and that PM_2.5_ could interact synergistically with heat wave and antagonistically with cold spell to increase the risk of pneumonia mortality, especially in men and individuals ≤ 80 years. Our findings emphasize urgent needs to address adverse effects of heat wave and high-level PM_2.5_ co-exposures on pneumonia mortality as a priority and highlight the importance to tailor policies on climate extremes and air pollution with consideration of the interactive effects between ETEs and PM_2.5_. Our findings provide novel epidemiological evidence on the complex interaction between ETEs and PM_2.5_. The results underscore urgent needs for targeted public health interventions and environmental policies to reduce co-exposures to heat wave and PM_2.5_. Given the substantial excess deaths associated with PM_2.5_ exposure, the identified antagonistic interaction of cold spell and PM_2.5_ further emphasizes the importance of prioritizing the reduction of PM_2.5_ exposure and promoting adaptive measures to prevent pneumonia deaths. Further studies are needed to explore the underlying biological mechanisms, examine other susceptible populations, and assess if similar interaction patterns exist for different respiratory diseases and in diverse climatic regions.

## Figures and Tables

**Table 1:** Characteristics of the study subjects in Jiangsu province, China, 2015–2022.

Characteristic	N (%)
Pneumonia deaths (case days), n	50,196
Control days, n	169,721
Age, mean (SD)	81.9 (15.3)
≤80, n (%)	14,316 (28.5)
>80, n (%)	35,880 (71.5)
Sex, n (%)	
Men	26,269 (52.3)
Women	23,925 (47.7)
Unknown	2 (0)
Season at death, n (%)	
Spring (March to May)	12,549 (25.0)
Summer (June to August)	10,299 (20.5)
Autumn (September to November)	10,763 (21.4)
Winter (December to February)	16,585 (33.0)

SD, standard deviation.

**Table 2:** Distribution of air pollutants and meteorological factors on case days and control days.

Exposure	On Case Days				On Control Days			
	Mean	P25	P50	P75	Mean	P25	P50	P75
PM_2.5_ (μg/m^3^)	49.0	26.4	40.8	62.2	48.5	26.1	40.0	61.6
O_3_ (μg/m^3^)	103.1	68.4	94.1	132.1	102.8	68.2	94.1	131.9
Heat index (°C)	15.7	5.6	14.2	24.0	15.7	5.7	14.4	24.0

P25—the 25th percentile; P50—the 50th percentile; P75—the 75th percentile; PM_2.5_—fine particulate matter; O_3_—ozone.

**Table 3:** Cumulative odds ratio of pneumonia mortality associated with exposure to heat wave, cold spell, and PM_2.5_^1^.

Heat Wave		Cold Spell		PM_2.5_^2^	
Definition	OR (95% CI)	Definition	OR (95% CI)	Definition	OR (95% CI)
P90_2d	1.23 (1.15, 1.32)	P10_2d	1.10 (1.04, 1.17)	P90/10_2d	1.016 (1.009, 1.024)
P90_3d	1.22 (1.14, 1.31)	P10_3d	1.10 (1.04, 1.17)	P90/10_3d	1.016 (1.008, 1.023)
P90_4d	1.23 (1.14, 1.32)	P10_4d	1.10 (1.03, 1.18)	P90/10_4d	1.015 (1.008, 1.023)
P92.5_2d	1.27 (1.18, 1.36)	P7.5_2d	1.11 (1.04, 1.18)	P92.5/7.5_2d	1.016 (1.008, 1.023)
P92.5_3d	1.25 (1.16, 1.35)	P7.5_3d	1.10 (1.02, 1.18)	P92.5/7.5_3d	1.015 (1.007, 1.022)
P92.5_4d	1.25 (1.16, 1.36)	P7.5_4d	1.11 (1.02, 1.20)	P92.5/7.5_4d	1.014 (1.007, 1.022)
P95_2d	1.36 (1.25, 1.48)	P5_2d	1.08 (1.002, 1.17)	P95/5_2d	1.015 (1.007, 1.022)
P95_3d	1.35 (1.24, 1.48)	P5_3d	1.08 (0.99, 1.18)	P95/5_3d	1.014 (1.007, 1.021)
P95_4d	1.35 (1.22, 1.48)	P5_4d	1.11 (1.002, 1.22)	P95/5_4d	1.013 (1.006, 1.021)
P97.5_2d	1.53 (1.36, 1.71)	P2.5_2d	1.16 (1.04, 1.28)	P97.5/2.5_2d	1.014 (1.007, 1.022)
P97.5_3d	1.60 (1.40, 1.81)	P2.5_3d	1.17 (1.03, 1.33)	P97.5/2.5_3d	1.014 (1.007, 1.022)
P97.5_4d	1.59 (1.38, 1.84)	P2.5_4d	1.18 (1.01, 1.38)	P97.5/2.5_4d	1.013 (1.006, 1.021)

PM_2.5_, fine particulate matter; OR, odds ratio; CI, confidence interval. ^1^ Lag structure up to 6 days for both heat wave and cold spell. ^2^ Cumulative association of PM_2.5_ (lag 0–6 day) exposure with pneumonia deaths were estimated by adjusting heat wave (P90_2d) and cold spell (P10_2d).

## Data Availability

The meteorological data are available at [http://data.cma.cn; accessed on 1 January 2024]. The air pollution data (CHAP dataset) are available at [https://weijing-rs.github.io; accessed on 1 January 2024]. The mortality data are not publicly available.

## Supplementary Materials

The following supporting information can be downloaded at: https://www.mdpi.com/article/10.3390/toxics13080702/s1.

## Abbreviations

The following abbreviations are used in this manuscript:

GBD	Global Burden of Diseases, Injuries, and Risk Factors Study
LRI	lower respiratory infections
YLLs	years of life lost
ETEs	extreme temperature events
PM_2.5_	fine particulate matter
ICD-10	International Statistical Classification of Diseases and Related Health Problems
HI	heat index
O_3_	ozone
CHAP	ChinaHighAirPollutants
R^2^	cross-validated coefficients of determination
RMSE	root-mean-square deviations
DLNM	distributed lag nonlinear model
*df*	degrees of freedom
OR	odds ratio
CI	confidence interval
RERI	relative excess risk due to interaction
AP	attributable proportion due to interaction
S	synergy index
eCI	empirical confidence interval
WHO	World Health Organization
AQGs	air quality guidelines
