# Supplementary material for: Opposite Interactive Effects of Heat Wave and Cold Spell with Fine Particulate Matter on Pneumonia Mortality
Source: Toxics. 2025 Aug 21;13(8):702. doi: 10.3390/toxics13080702 (PMC12390074; doi:10.3390/toxics13080702)
Supplement: Supplementary file 1 [file toxics-13-00702-s001.zip › toxics-3764486-supplementary.pdf]

Opposite interactive effects of heat wave and cold spell with fine particulate matter on pneumonia mortality

Yi Zheng[1,†], Ruijun Xu[1,†], Yuling Chen[1,2], Yingxin Li[1], Yuxin Bi[1], Xiaohong Jia[1], Sirong Wang[1], Lu Luo[1], Jing Wei[3,] Rui Wang[4], Chunxiang Shi[5], Ziquan Lv[6], Suli Huang[7], Gongbo Chen[8], Hong Sun[9], Bochao Sun[10], Nongping Feng[11,*], Yuewei Liu[1,*]

Number of pages: 31

Number of tables: 21

Number of figures: 3

Table S1. The threshold of heat wave, cold spell are defined by the heat index, air temperature, and apparent temperature

Table S2. The number of pneumonia deaths during different exposure levels of heat wave, cold spell, and $PM_{2.5}$

Table S3. Cumulative association of exposure to ETEs and high-level $PM_{2.5}$ on pneumonia mortality

Table S4. Additive interactive effects of exposure to ETEs and high-level $PM_{2.5}$ on pneumonia mortality

Table S5. Excess fraction and number of excess deaths of pneumonia from exposure to ETEs and high-level $PM_{2.5}$

Table S6. Subgroup analyses: cumulative association of ETE exposure with pneumonia deaths in women and men

Table S7. Subgroup analyses: cumulative association of $PM_{2.5}$ exposure with pneumonia deaths in women and men

Table S8. Subgroup analyses: additive interactive effects of exposure to ETEs and high-level $PM_{2.5}$ on pneumonia mortality in women and men

Table S9. Subgroup analyses: cumulative association of ETE exposure with pneumonia deaths in individuals aged > 80 years and ≤ 80 years

Table S10. Subgroup analyses: cumulative association of $PM_{2.5}$ exposure with pneumonia deaths in individuals aged > 80 years and ≤ 80 years

Table S11. Subgroup analyses: additive interactive effects of exposure to ETEs and $PM_{2.5}$ on pneumonia mortality in individuals aged > 80 years and ≤ 80 years

Table S12. Sensitivity analyses: odds ratio (95% CI) of mortality from pneumonia associated with exposure to heat wave, cold spell, and $PM_{2.5}$ with O_3 adjusted

Table S13. Sensitivity analyses: relative excess risk due to interaction of exposure to ETEs and high-level $PM_{2.5}$ on mortality from pneumonia with O_3 adjusted

Table S14. Sensitivity analyses: odds ratio (95% CI) of mortality from pneumonia associated with exposure to heat wave, cold spell, and $PM_{2.5}$ in different season

Table S15. Sensitivity analyses: odds ratio (95% CI) of mortality from pneumonia associated with exposure to heat wave, cold spell, and $PM_{2.5}$ with 37.5 $\mu g/m^3$ as threshold value

Table S16. Sensitivity analyses: relative excess risk due to interaction of exposure to ETEs and high-level $PM_{2.5}$ with 37.5 μg/m^3 as threshold value on mortality from pneumonia

Table S17. Sensitivity analyses: excess fraction and number of excess deaths of pneumonia from exposure to ETEs and high-level $PM_{2.5}$ with 37.5 μg/m^3 as threshold value

Table S18. Sensitivity analyses (using air temperature): odds ratio (95% CI) of mortality from pneumonia associated with exposure to heat wave, cold spell, and $PM_{2.5}$

Table S19. Sensitivity analyses (using air temperature): relative excess risk due to interaction of exposure to ETEs and high-level $PM_{2.5}$

Table S20. Sensitivity analyses (using apparent temperature): odds ratio (95% CI) of mortality from pneumonia associated with exposure to heat wave, cold spell, and $PM_{2.5}$

Table S21. Sensitivity analyses (using apparent temperature): relative excess risk due to interaction of exposure to ETEs and high-level $PM_{2.5}$

Figure S1. Exposure-response curve of the association between exposure to $PM_{2.5}$ and pneumonia mortality

Figure S2. Overall lag structure for the association of ETE exposure with pneumonia mortality in Jiangsu province, China, during 2015-2022

Figure S3. Overall lag structure for the association of $PM_{2.5}$ exposure with pneumonia mortality in Jiangsu province, China, during 2015-2022

Table S1. The threshold of heat wave, cold spell are defined by the heat index, air temperature, and apparent temperature

	Heat index, °C	Air temperature, °C	Apparent temperature, °C
Heat wave			
P90	31.6	27.8	32.2
P92.5	33.6	28.5	33.4
P95	35.6	29.5	34.8
P97.5	38.7	30.5	36.5
Cold spell			
P10	3.2	3.9	0.1
P7.5	2.5	3.1	-0.9
P5	1.7	2.1	-2.1
P2.5	0.4	0.7	-4.0

Table S2. The number of pneumonia deaths during different exposure levels of heat wave, cold spell, and $PM_{2.5}$[a,b]

Heat wave	All	Co-exposure	Cold spell	All	Co-exposure
	Number	Number		Number	Number
P90_2d	3,728	465	P10_2d	5,221	3,545
P90_3d	3,226	405	P10_3d	3,837	2,682
P90_4d	2,904	382	P10_4d	2,924	2,084
P92.5_2d	2,816	411	P7.5_2d	3,738	2,502
P92.5_3d	2,402	378	P7.5_3d	2,587	1,816
P92.5_4d	2,058	352	P7.5_4d	1,865	1,349
P95_2d	1,900	350	P5_2d	2,422	1,598
P95_3d	1,539	316	P5_3d	1,648	1,150
P95_4d	1,288	294	P5_4d	1,209	886
P97.5_2d	969	272	P2.5_2d	1,253	808
P97.5_3d	759	243	P2.5_3d	855	580
P97.5_4d	583	200	P2.5_4d	545	412

[a]$PM_{2.5}$, fine particulate matter. [b]The stratification of $PM_{2.5}$ is based on a threshold value of 40.8 μg/m^3.

Table S3. Cumulative association of exposure to ETEs and high-level $PM_{2.5}$ on pneumonia mortality[a]

ETEs	$PM_{2.5}$	ETEs	Co-exposure
	OR_{01} (95% CI)	OR_{10} (95% CI)	OR_{11} (95% CI)
Heat wave			
P90_2d	1.14 (1.08, 1.20)	1.17 (1.09, 1.27)	1.71 (1.41, 2.08)
P90_3d	1.13 (1.07, 1.18)	1.14 (1.06, 1.24)	1.82 (1.49, 2.22)
P90_4d	1.12 (1.06, 1.18)	1.15 (1.06, 1.25)	1.81 (1.48, 2.22)
P92.5_2d	1.13 (1.07, 1.19)	1.18 (1.08, 1.29)	1.88 (1.54, 2.30)
P92.5_3d	1.12 (1.06, 1.18)	1.15 (1.05, 1.25)	1.95 (1.58, 2.40)
P92.5_4d	1.11 (1.06, 1.17)	1.13 (1.03, 1.24)	2.02 (1.63, 2.50)
P95_2d	1.12 (1.06, 1.18)	1.26 (1.14, 1.39)	1.97 (1.58, 2.44)
P95_3d	1.12 (1.06, 1.17)	1.22 (1.09, 1.36)	2.04 (1.62, 2.55)
P95_4d	1.11 (1.05, 1.16)	1.17 (1.04, 1.32)	2.13 (1.69, 2.69)
P97.5_2d	1.11 (1.06, 1.17)	1.36 (1.18, 1.57)	2.11 (1.65, 2.69)
P97.5_3d	1.11 (1.05, 1.16)	1.38 (1.17, 1.63)	2.31 (1.79, 3.00)
P97.5_4d	1.10 (1.05, 1.15)	1.31 (1.08, 1.58)	2.56 (1.91, 3.43)
Cold spell			
P10_2d	1.14 (1.08, 1.20)	1.25 (1.10, 1.42)	1.19 (1.09, 1.30)
P10_3d	1.13 (1.07, 1.18)	1.29 (1.10, 1.50)	1.17 (1.07, 1.28)
P10_4d	1.12 (1.06, 1.18)	1.30 (1.09, 1.56)	1.17 (1.06, 1.29)
P7.5_2d	1.13 (1.07, 1.19)	1.26 (1.09, 1.45)	1.18 (1.07, 1.30)
P7.5_3d	1.12 (1.06, 1.18)	1.41 (1.17, 1.69)	1.12 (1.01, 1.24)
P7.5_4d	1.11 (1.06, 1.17)	1.60 (1.27, 2.02)	1.09 (0.96, 1.22)
P5_2d	1.12 (1.06, 1.18)	1.35 (1.13, 1.60)	1.09 (0.97, 1.22)
P5_3d	1.12 (1.06, 1.17)	1.61 (1.26, 2.05)	1.03 (0.90, 1.17)
P5_4d	1.11 (1.05, 1.16)	1.93 (1.40, 2.65)	1.02 (0.88, 1.18)
P2.5_2d	1.11 (1.06, 1.17)	1.42 (1.13, 1.79)	1.17 (0.996, 1.37)
P2.5_3d	1.11 (1.05, 1.16)	1.41 (0.99, 1.99)	1.26 (1.04, 1.53)
P2.5_4d	1.10 (1.05, 1.15)	1.59 (0.92, 2.74)	1.26 (1.02, 1.57)

[a]ETEs, extreme temperature events; $PM_{2.5}$, fine particulate matter; CI, confidence interval; OR_{11}, co-exposure to ETEs and high-level $PM_{2.5}$; OR_{10}, exposure to ETEs; OR_{01}, exposure to high-level $PM_{2.5}$.

Table S4. Additive interactive effects of exposure to ETEs and high-level $PM_{2.5}$ on pneumonia mortality[a,b,c]

ETEs	RERI (95% CI)	AP (95% CI)	S (95% CI)
Heat wave			
P90_2d	0.40 (0.06, 0.76)	0.23 (0.05, 0.38)	2.29 (1.19, 4.04)
P90_3d	0.55 (0.19, 0.95)	0.30 (0.13, 0.44)	3.04 (1.58, 5.65)
P90_4d	0.55 (0.16, 0.97)	0.30 (0.12, 0.45)	3.04 (1.56, 6.02)
P92.5_2d	0.58 (0.17, 1.00)	0.31 (0.11, 0.44)	2.88 (1.48, 5.26)
P92.5_3d	0.68 (0.27, 1.17)	0.35 (0.17, 0.49)	3.54 (1.82, 7.23)
P92.5_4d	0.78 (0.36, 1.30)	0.39 (0.22, 0.52)	4.25 (2.14, 9.93)
P95_2d	0.59 (0.16, 1.10)	0.30 (0.10, 0.46)	2.56 (1.33, 4.91)
P95_3d	0.70 (0.21, 1.27)	0.34 (0.13, 0.50)	3.10 (1.47, 6.53)
P95_4d	0.85 (0.37, 1.45)	0.40 (0.22, 0.54)	4.01 (2.01, 8.76)
P97.5_2d	0.63 (0.08, 1.26)	0.30 (0.05, 0.48)	2.33 (1.13, 4.76)
P97.5_3d	0.83 (0.18, 1.55)	0.36 (0.10, 0.53)	2.70 (1.31, 5.61)
P97.5_4d	1.16 (0.41, 2.09)	0.45 (0.21, 0.62)	3.84 (1.70, 10.47)
Cold spell			
P10_2d	-0.20 (-0.40, -0.03)	-0.17 (-0.35, -0.02)	0.49 (0.23, 0.89)
P10_3d	-0.24 (-0.50, -0.01)	-0.20 (-0.44, -0.004)	0.42 (0.16, 0.98)
P10_4d	-0.26 (-0.55, 0.01)	-0.22 (-0.50, 0.01)	0.39 (0.12, 1.05)
P7.5_2d	-0.20 (-0.43, 0.01)	-0.17 (-0.38, 0.01)	0.47 (0.17, 1.04)
P7.5_3d	-0.41 (-0.75, -0.11)	-0.36 (-0.70, -0.10)	0.23 (0.02, 0.61)
P7.5_4d	-0.63 (-1.09, -0.22)	-0.58 (-1.09, -0.20)	0.12 (-0.05, 0.44)
P5_2d	-0.38 (-0.68, -0.10)	-0.35 (-0.66, -0.09)	0.19 (-0.05, 0.61)
P5_3d	-0.70 (-1.18, -0.25)	-0.68 (-1.26, -0.23)	0.04 (-0.13, 0.32)
P5_4d	-1.02 (-1.78, -0.38)	-1.00 (-1.93, -0.35)	0.02 (-0.11, 0.27)
P2.5_2d	-0.37 (-0.82, 0.03)	-0.31 (-0.80, 0.02)	0.32 (-0.02, 1.15)
P2.5_3d	-0.25 (-0.97, 0.32)	-0.20 (-0.88, 0.22)	0.51 (0.03, 4.11)
P2.5_4d	-0.42 (-1.69, 0.38)	-0.33 (-1.52, 0.28)	0.39 (-1.73, 2.43)

[a]ETEs, extreme temperature events; $PM_{2.5}$, fine particulate matter; CI, confidence interval; RERI, relative excess risk due to interaction; AP, attributable proportion due to interaction; S, synergy index. [b]The stratification of $PM_{2.5}$ is based on a threshold value of 40.8 μg/m^3. [c]RERI and AP greater than 0, and S greater than 1 indicate a positive interaction (synergistic effect), while RERI and AP, and S smaller than 1 indicate a negative interaction.

1 **Table S5.** Excess fraction and number of excess deaths of pneumonia from exposure to ETEs and high-level $PM_{2.5}$[a,b]

Definition	Number of excess death (N)			Excess fraction (%)		
	$PM_{2.5}$	ETEs	Co-exposure	$PM_{2.5}$	ETEs	Co-exposure
Heat wave						
P90_2d	2,561 (1,501, 3,551)	489 (258, 706)	211 (135, 276)	5.10 (2.87, 6.96)	0.97 (0.53, 1.40)	0.42 (0.28, 0.55)
P90_3d	2,461 (1,350, 3,550)	359 (145, 556)	209 (145, 269)	4.90 (2.70, 7.00)	0.71 (0.28, 1.09)	0.42 (0.29, 0.53)
P90_4d	2,417 (1,346, 3,448)	328 (137, 501)	193 (132, 245)	4.81 (2.64, 6.91)	0.65 (0.24, 0.99)	0.38 (0.26, 0.49)
P92.5_2d	2,501 (1,433, 3,500)	373 (177, 552)	214 (146, 267)	4.98 (2.98, 7.05)	0.74 (0.39, 1.09)	0.43 (0.31, 0.54)
P92.5_3d	2,475 (1,453, 3,530)	262 (97, 431)	207 (141, 259)	4.93 (2.74, 6.89)	0.52 (0.20, 0.81)	0.41 (0.29, 0.51)
P92.5_4d	2,383 (1,269, 3,520)	193 (41, 326)	201 (144, 249)	4.75 (2.63, 6.98)	0.38 (0.08, 0.66)	0.40 (0.28, 0.49)
P95_2d	2,495 (1,461, 3,566)	328 (189, 449)	193 (133, 244)	4.97 (2.87, 7.07)	0.65 (0.39, 0.91)	0.38 (0.27, 0.48)
P95_3d	2,462 (1,396, 3,552)	227 (108, 338)	180 (127, 222)	4.91 (2.69, 7.00)	0.45 (0.24, 0.67)	0.36 (0.26, 0.45)
P95_4d	2,340 (1,200, 3,514)	151 (45, 251)	172 (126, 209)	4.66 (2.52, 6.63)	0.30 (0.08, 0.49)	0.34 (0.24, 0.42)
P97.5_2d	2,439 (1,325, 3,576)	198 (111, 273)	159 (110, 198)	4.86 (2.57, 6.86)	0.39 (0.22, 0.55)	0.32 (0.22, 0.39)
P97.5_3d	2,317 (1,147, 3,409)	149 (78, 214)	151 (108, 186)	4.62 (2.52, 6.79)	0.30 (0.15, 0.42)	0.30 (0.22, 0.37)
P97.5_4d	2,219 (1,112, 3,195)	93 (30, 148)	136 (100, 167)	4.42 (2.15, 6.67)	0.19 (0.06, 0.30)	0.27 (0.20, 0.33)
Cold spell						
P10_2d	2,561 (1,628, 3,561)	386 (156, 599)	601 (299, 871)	5.10 (3.01, 7.17)	0.77 (0.35, 1.20)	1.20 (0.61, 1.73)
P10_3d	2,461 (1,444, 3,525)	287 (110, 437)	411 (166, 620)	4.90 (2.82, 6.79)	0.57 (0.23, 0.89)	0.82 (0.36, 1.22)
P10_4d	2,417 (1,323, 3,487)	218 (64, 355)	310 (128, 489)	4.81 (2.68, 6.88)	0.43 (0.16, 0.71)	0.62 (0.23, 0.99)
P7.5_2d	2,501 (1,509, 3,552)	294 (114, 468)	396 (153, 612)	4.98 (2.86, 7.00)	0.59 (0.23, 0.94)	0.79 (0.35, 1.22)
P7.5_3d	2,475 (1,388, 3,508)	263 (113, 397)	199 (-2, 384)	4.93 (2.83, 7.03)	0.52 (0.25, 0.77)	0.40 (0.02, 0.78)

Definition	Number of excess death (N)			Excess fraction (%)		
	$PM_{2.5}$	ETEs	Co-exposure	$PM_{2.5}$	ETEs	Co-exposure
P7.5_4d	2,383 (1,371, 3,395)	234 (115, 340)	110 (-59, 266)	4.75 (2.67, 6.78)	0.47 (0.23, 0.67)	0.22 (-0.09, 0.51)
P5_2d	2,495 (1,392, 3,488)	256 (96, 403)	139 (-52, 311)	4.97 (2.75, 6.96)	0.51 (0.23, 0.80)	0.28 (-0.09, 0.64)
P5_3d	2,462 (1,340, 3,462)	239 (130, 338)	31 (-137, 179)	4.91 (2.74, 6.93)	0.48 (0.22, 0.70)	0.06 (-0.27, 0.37)
P5_4d	2,340 (1,251, 3,455)	205 (110, 285)	18 (-119, 153)	4.66 (2.54, 6.77)	0.41 (0.20, 0.58)	0.04 (-0.25, 0.27)
P2.5_2d	2,439 (1,301, 3,579)	160 (48, 253)	122 (-1, 242)	4.86 (2.55, 7.15)	0.32 (0.10, 0.52)	0.24 (0.00, 0.47)
P2.5_3d	2,317 (1,144, 3,399)	87 (2, 172)	129 (23, 226)	4.62 (2.50, 6.79)	0.17 (0.00, 0.33)	0.26 (0.04, 0.43)
P2.5_4d	2,219 (951, 3,272)	54 (-14, 113)	95 (10, 170)	4.42 (2.18, 6.44)	0.11 (-0.03, 0.23)	0.19 (-0.01, 0.34)

[a]ETEs, extreme temperature events; $PM_{2.5}$, fine particulate matter. [b]The stratification of $PM_{2.5}$ is based on a threshold value of 40.8 μg/m^3.

Table S6. Subgroup analyses: cumulative association of ETE exposure with pneumonia deaths in women and men[a]

Definition	Women	Men	*P* value[b]
	OR (95% CI)	OR (95% CI)	
Heat wave			
P90_2d	1.28 (1.16, 1.42)	1.18 (1.07, 1.30)	0.27
P90_3d	1.27 (1.15, 1.41)	1.17 (1.06, 1.30)	0.26
P90_4d	1.27 (1.15, 1.41)	1.19 (1.08, 1.32)	0.39
P92.5_2d	1.33 (1.19, 1.47)	1.21 (1.09, 1.34)	0.24
P92.5_3d	1.30 (1.17, 1.45)	1.21 (1.09, 1.34)	0.32
P92.5_4d	1.28 (1.14, 1.43)	1.23 (1.10, 1.37)	0.60
P95_2d	1.48 (1.31, 1.66)	1.26 (1.12, 1.42)	0.07
P95_3d	1.46 (1.28, 1.65)	1.26 (1.11, 1.43)	0.11
P95_4d	1.42 (1.24, 1.63)	1.27 (1.11, 1.45)	0.25
P97.5_2d	1.66 (1.41, 1.94)	1.40 (1.20, 1.64)	0.15
P97.5_3d	1.73 (1.44, 2.07)	1.47 (1.23, 1.76)	0.22
P97.5_4d	1.71 (1.39, 2.11)	1.49 (1.21, 1.83)	0.35
Cold spell			
P10_2d	1.13 (1.04, 1.23)	1.08 (0.996, 1.17)	0.48
P10_3d	1.13 (1.03, 1.23)	1.08 (0.99, 1.17)	0.45
P10_4d	1.13 (1.03, 1.24)	1.08 (0.98, 1.19)	0.53
P7.5_2d	1.14 (1.04, 1.25)	1.08 (0.98, 1.18)	0.42
P7.5_3d	1.14 (1.03, 1.26)	1.06 (0.96, 1.17)	0.32
P7.5_4d	1.15 (1.02, 1.29)	1.07 (0.95, 1.20)	0.40
P5_2d	1.14 (1.02, 1.27)	1.03 (0.92, 1.15)	0.21
P5_3d	1.13 (1.002, 1.28)	1.03 (0.92, 1.17)	0.30
P5_4d	1.15 (0.996, 1.32)	1.07 (0.93, 1.23)	0.49
P2.5_2d	1.18 (1.02, 1.37)	1.13 (0.98, 1.31)	0.69
P2.5_3d	1.20 (1.001, 1.43)	1.15 (0.96, 1.37)	0.74
P2.5_4d	1.27 (1.02, 1.57)	1.11 (0.89, 1.39)	0.41

[a]ETEs, extreme temperature events; OR, odds ratio; CI, confidence interval. [b]Estimated by the 2-sample *z* test.

Table S7. Subgroup analyses: cumulative association of $PM_{2.5}$ exposure with pneumonia deaths in women and men[a]

Definition	Women	Men	*P* value[b]
	OR (95% CI)	OR (95% CI)	
P90/10_2d	1.023 (1.012, 1.034)	1.009 (0.999, 1.020)	0.08
P90/10_3d	1.022 (1.011, 1.033)	1.009 (0.999, 1.020)	0.10
P90/10_4d	1.021 (1.010, 1.032)	1.010 (0.999, 1.020)	0.13
P92.5/7.5_2d	1.022 (1.012, 1.033)	1.009 (0.999, 1.020)	0.09
P92.5/7.5_3d	1.021 (1.011, 1.032)	1.009 (0.998, 1.019)	0.10
P92.5/7.5_4d	1.020 (1.009, 1.031)	1.008 (0.998, 1.019)	0.12
P95/5_2d	1.021 (1.010, 1.032)	1.008 (0.998, 1.019)	0.09
P95/5_3d	1.020 (1.009, 1.030)	1.009 (0.998, 1.019)	0.14
P95/5_4d	1.019 (1.008, 1.030)	1.008 (0.998, 1.019)	0.15
P97.5/2.5_2d	1.020 (1.010, 1.031)	1.008 (0.998, 1.019)	0.12
P97.5/2.5_3d	1.020 (1.009, 1.030)	1.009 (0.999, 1.019)	0.16
P97.5/2.5_4d	1.019 (1.008, 1.029)	1.009 (0.998, 1.019)	0.19

[a]$PM_{2.5}$, fine particulate matter; OR, odds ratio; CI, confidence interval. [b]Estimated by the 2-sample *z* test.

Table S8. Subgroup analyses: additive interactive effects of exposure to ETEs and high-level $PM_{2.5}$ on pneumonia mortality in women and men[a,b,c]

Definition	Men	Women	Men	Women	Men	Women
	RERI (95% CI)	RERI (95% CI)	AP (95% CI)	AP (95% CI)	S (95% CI)	S (95% CI)
Heat wave						
P90_2d	0.62 (0.13, 1.15)	0.16 (-0.28, 0.64)	0.35 (0.09, 0.50)	0.10 (-0.22, 0.32)	4.57 (1.42, 17.78)	1.33 (0.47, 2.79)
P90_3d	0.71 (0.19, 1.33)	0.36 (-0.16, 0.93)	0.38 (0.13, 0.56)	0.20 (-0.12, 0.41)	5.97 (1.05, 37.36)	1.86 (0.69, 3.87)
P90_4d	0.68 (0.15, 1.33)	0.38 (-0.08, 0.99)	0.37 (0.11, 0.55)	0.22 (-0.06, 0.43)	5.35 (1.28, 27.36)	1.97 (0.81, 4.53)
P92.5_2d	0.87 (0.27, 1.53)	0.26 (-0.22, 0.84)	0.43 (0.18, 0.58)	0.15 (-0.17, 0.37)	6.49 (1.84, 39.44)	1.53 (0.56, 3.33)
P92.5_3d	0.93 (0.32, 1.69)	0.41 (-0.15, 1.04)	0.45 (0.20, 0.61)	0.22 (-0.11, 0.43)	7.59 (-33.86, 64.72)	1.98 (0.70, 4.26)
P92.5_4d	1.01 (0.38, 1.79)	0.54 (-0.08, 1.19)	0.47 (0.24, 0.62)	0.28 (-0.05, 0.48)	8.03 (-27.96, 49.48)	2.52 (0.82, 6.04)
P95_2d	0.92 (0.30, 1.72)	0.21 (-0.38, 0.89)	0.44 (0.20, 0.61)	0.11 (-0.29, 0.36)	6.17 (1.64, 42.30)	1.33 (0.47, 2.85)
P95_3d	1.01 (0.32, 1.93)	0.36 (-0.30, 1.09)	0.47 (0.21, 0.64)	0.19 (-0.22, 0.43)	7.56 (-21.94, 69.30)	1.65 (0.58, 3.74)
P95_4d	1.17 (0.41, 2.09)	0.52 (-0.19, 1.37)	0.51 (0.25, 0.67)	0.26 (-0.14, 0.51)	10.37 (-96.25, 83.83)	2.12 (0.68, 5.57)
P97.5_2d	1.04 (0.24, 1.95)	0.16 (-0.63, 1.01)	0.46 (0.15, 0.64)	0.08 (-0.45, 0.39)	5.63 (-9.40, 37.34)	1.20 (0.38, 2.84)
P97.5_3d	0.96 (0.06, 1.95)	0.54 (-0.32, 1.58)	0.43 (0.04, 0.63)	0.24 (-0.20, 0.51)	4.35 (0.88, 24.72)	1.74 (0.63, 4.29)
P97.5_4d	1.23 (0.17, 2.53)	0.95 (-0.05, 2.27)	0.50 (0.11, 0.71)	0.37 (-0.03, 0.62)	6.05 (-37.90, 63.47)	2.59 (0.93, 8.19)
Cold spell						
P10_2d	-0.18 (-0.47, 0.08)	-0.21 (-0.53, 0.04)	-0.17 (-0.45, 0.07)	-0.16 (-0.42, 0.03)	0.34 (-0.18, 1.90)	0.59 (0.30, 1.14)
P10_3d	-0.20 (-0.53, 0.08)	-0.28 (-0.67, 0.06)	-0.18 (-0.53, 0.08)	-0.22 (-0.56, 0.05)	0.30 (-0.26, 2.07)	0.49 (0.19, 1.23)
P10_4d	-0.22 (-0.60, 0.14)	-0.31 (-0.78, 0.12)	-0.20 (-0.60, 0.13)	-0.25 (-0.65, 0.09)	0.30 (-0.56, 2.59)	0.45 (0.14, 1.51)
P7.5_2d	-0.20 (-0.51, 0.11)	-0.21 (-0.58, 0.15)	-0.18 (-0.50, 0.09)	-0.16 (-0.48, 0.10)	0.28 (-0.41, 1.81)	0.59 (0.25, 1.60)

Definition	Men	Women	Men	Women	Men	Women
	RERI (95% CI)	RERI (95% CI)	AP (95% CI)	AP (95% CI)	S (95% CI)	S (95% CI)
P7.5_3d	-0.46 (-0.92, -0.08)	-0.35 (-0.86, 0.11)	-0.45 (-0.97, -0.07)	-0.28 (-0.75, 0.08)	0.01 (-0.38, 0.58)	0.42 (0.10, 1.34)
P7.5_4d	-0.78 (-1.49, -0.27)	-0.45 (-1.16, 0.14)	-0.81 (-1.71, -0.24)	-0.37 (-1.02, 0.10)	-0.05 (-0.27, 0.29)	0.34 (0.05, 1.62)
P5_2d	-0.40 (-0.83, -0.01)	-0.34 (-0.84, 0.08)	-0.42 (-0.91, -0.01)	-0.27 (-0.74, 0.06)	-0.09 (-0.70, 0.73)	0.41 (0.06, 1.27)
P5_3d	-0.77 (-1.54, -0.22)	-0.60 (-1.38, -0.03)	-0.85 (-1.89, -0.22)	-0.52 (-1.36, -0.02)	-0.14 (-0.55, 0.27)	0.21 (-0.04, 0.92)
P5_4d	-1.47 (-2.81, -0.55)	-0.59 (-1.64, 0.20)	-1.69 (-3.64, -0.55)	-0.49 (-1.60, 0.15)	-0.10 (-0.27, 0.08)	0.25 (-0.04, 1.82)
P2.5_2d	-0.36 (-0.97, 0.20)	-0.37 (-1.05, 0.26)	-0.33 (-1.06, 0.16)	-0.29 (-0.95, 0.16)	0.18 (-0.53, 2.32)	0.42 (0.01, 1.91)
P2.5_3d	-0.46 (-1.50, 0.33)	-0.02 (-1.04, 0.73)	-0.42 (-1.60, 0.24)	-0.01 (-0.91, 0.44)	0.19 (-1.24, 2.70)	0.97 (-7.82, 8.95)
P2.5_4d	-0.75 (-2.90, 0.41)	-0.03 (-1.92, 1.14)	-0.71 (-3.11, 0.33)	-0.02 (-1.56, 0.58)	0.07 (-1.92, 2.16)	0.94 (-14.77, 10.46)

[a]ETEs, extreme temperature events; $PM_{2.5}$, fine particulate matter; CI, confidence interval; RERI, relative excess risk due to interaction; AP, attributable proportion due to interaction; S, synergy index. [b]The stratification of $PM_{2.5}$ is based on a threshold value of 40.8 μg/m^3. [c]RERI and AP greater than 0, and S greater than 1 indicate a positive interaction (synergistic effect), while RERI and AP, and S smaller than 1 indicate a negative interaction.

Table S9. Subgroup analyses: cumulative association of ETE exposure with pneumonia deaths in individuals aged > 80 years and ≤ 80 years[a]

Definition	> 80	≤ 80	*P* value[b]
	OR (95% CI)	OR (95% CI)	
Heat wave			
P90_2d	1.10 (0.97, 1.25)	1.29 (1.18, 1.40)	0.05
P90_3d	1.14 (0.998, 1.30)	1.26 (1.16, 1.37)	0.20
P90_4d	1.16 (1.01, 1.32)	1.26 (1.16, 1.37)	0.30
P92.5_2d	1.18 (1.03, 1.36)	1.30 (1.19, 1.42)	0.24
P92.5_3d	1.20 (1.04, 1.38)	1.28 (1.17, 1.40)	0.47
P92.5_4d	1.21 (1.04, 1.40)	1.27 (1.16, 1.39)	0.60
P95_2d	1.23 (1.05, 1.44)	1.42 (1.29, 1.57)	0.12
P95_3d	1.22 (1.03, 1.45)	1.41 (1.27, 1.56)	0.17
P95_4d	1.26 (1.05, 1.51)	1.38 (1.23, 1.54)	0.40
P97.5_2d	1.32 (1.07, 1.63)	1.61 (1.41, 1.84)	0.12
P97.5_3d	1.35 (1.06, 1.72)	1.69 (1.45, 1.96)	0.12
P97.5_4d	1.34 (1.01, 1.76)	1.69 (1.42, 2.01)	0.16
Cold spell			
P10_2d	1.08 (0.97, 1.20)	1.11 (1.04, 1.19)	0.64
P10_3d	1.11 (0.99, 1.25)	1.10 (1.02, 1.18)	0.87
P10_4d	1.13 (0.998, 1.28)	1.09 (1.01, 1.18)	0.65
P7.5_2d	1.10 (0.98, 1.25)	1.11 (1.03, 1.20)	0.95
P7.5_3d	1.11 (0.97, 1.27)	1.09 (1.002, 1.19)	0.86
P7.5_4d	1.13 (0.97, 1.31)	1.10 (0.99, 1.21)	0.76
P5_2d	1.07 (0.93, 1.23)	1.08 (0.99, 1.19)	0.88
P5_3d	1.06 (0.91, 1.24)	1.09 (0.98, 1.21)	0.80
P5_4d	1.05 (0.87, 1.25)	1.13 (1.003, 1.27)	0.49
P2.5_2d	1.02 (0.85, 1.24)	1.22 (1.08, 1.38)	0.14
P2.5_3d	0.97 (0.77, 1.23)	1.27 (1.09, 1.47)	0.06
P2.5_4d	0.91 (0.68, 1.22)	1.32 (1.10, 1.58)	0.03

[a]ETEs, extreme temperature events; OR, odds ratio; CI, confidence interval. [b]Estimated by the 2-sample z test.

Table S10. Subgroup analyses: Cumulative association of $PM_{2.5}$ exposure with pneumonia deaths in individuals aged > 80 years and ≤ 80 years[a]

Definition	> 80	≤ 80	*P* value[b]
	OR (95% CI)	OR (95% CI)	
P90/10_2d	1.018 (1.004, 1.032)	1.015 (1.006, 1.024)	0.74
P90/10_3d	1.017 (1.003, 1.031)	1.015 (1.006, 1.024)	0.80
P90/10_4d	1.017 (1.003, 1.031)	1.015 (1.006, 1.023)	0.77
P92.5/7.5_2d	1.018 (1.004, 1.032)	1.015 (1.006, 1.024)	0.70
P92.5/7.5_3d	1.017 (1.003, 1.031)	1.014 (1.005, 1.023)	0.73
P92.5/7.5_4d	1.016 (1.002, 1.030)	1.013 (1.005, 1.022)	0.72
P95/5_2d	1.017 (1.003, 1.032)	1.013 (1.005, 1.022)	0.63
P95/5_3d	1.016 (1.002, 1.030)	1.013 (1.004, 1.022)	0.71
P95/5_4d	1.016 (1.002, 1.030)	1.012 (1.004, 1.021)	0.69
P97.5/2.5_2d	1.018 (1.004, 1.032)	1.013 (1.004, 1.022)	0.54
P97.5/2.5_3d	1.018 (1.004, 1.032)	1.013 (1.004, 1.022)	0.51
P97.5/2.5_4d	1.019 (1.004, 1.033)	1.012 (1.003, 1.020)	0.41

[a]$PM_{2.5}$, fine particulate matter; OR, odds ratio; CI, confidence interval. [b]Estimated by the 2-sample *z* test.

Table S11. Subgroup analyses: additive interactive effects of exposure to ETEs and $PM_{2.5}$ on pneumonia mortality in individuals aged > 80 years and ≤ 80 years[a,b,c]

Definition	> 80	≤ 80	> 80	≤ 80	> 80	≤ 80
	RERI (95% CI)	RERI (95% CI)	AP (95% CI)	AP (95% CI)	S (95% CI)	S (95% CI)
Heat wave						
P90_2d	0.31 (-0.08, 0.73)	0.59 (-0.05, 1.35)	0.19 (-0.05, 0.36)	0.34 (-0.05, 0.56)	1.83 (0.84, 3.39)	4.45 (-31.22, 31.93)
P90_3d	0.46 (0.05, 0.91)	0.76 (0.08, 1.67)	0.26 (0.03, 0.42)	0.39 (0.06, 0.60)	2.45 (1.12, 5.06)	5.43 (-25.90, 43.70)
P90_4d	0.43 (0.04, 0.90)	0.82 (0.03, 1.75)	0.25 (0.03, 0.42)	0.41 (0.03, 0.62)	2.37 (1.10, 5.08)	6.03 (-30.67, 52.56)
P92.5_2d	0.49 (0.05, 1.00)	0.77 (0.08, 1.67)	0.27 (0.04, 0.44)	0.39 (0.06, 0.60)	2.42 (1.13, 4.98)	4.56 (0.92, 22.42)
P92.5_3d	0.58 (0.14, 1.12)	0.92 (0.17, 1.96)	0.31 (0.10, 0.48)	0.44 (0.12, 0.63)	2.97 (1.40, 7.05)	5.66 (0.80, 48.36)
P92.5_4d	0.69 (0.18, 1.26)	1.03 (0.13, 2.15)	0.35 (0.12, 0.51)	0.46 (0.10, 0.66)	3.63 (1.54, 8.99)	6.60 (-24.01, 55.95)
P95_2d	0.41 (-0.06, 0.98)	1.07 (0.22, 2.41)	0.22 (-0.04, 0.42)	0.47 (0.15, 0.68)	1.89 (0.89, 3.95)	6.15 (0.92, 39.75)
P95_3d	0.52 (0.03, 1.17)	1.18 (0.27, 2.50)	0.27 (0.02, 0.47)	0.50 (0.18, 0.70)	2.28 (1.05, 5.00)	8.04 (-55.51, 113.95)
P95_4d	0.73 (0.15, 1.47)	1.19 (0.21, 2.74)	0.36 (0.10, 0.55)	0.50 (0.13, 0.73)	3.25 (1.37, 8.37)	7.98 (-71.85, 70.83)
P97.5_2d	0.38 (-0.25, 1.13)	1.26 (0.23, 2.85)	0.19 (-0.16, 0.44)	0.52 (0.15, 0.73)	1.63 (0.67, 3.71)	7.85 (-75.00, 111.08)
P97.5_3d	0.66 (-0.02, 1.54)	1.18 (0.06, 2.73)	0.29 (-0.01, 0.52)	0.50 (0.04, 0.73)	2.09 (0.97, 4.86)	6.93 (-67.61, 76.44)
P97.5_4d	1.07 (0.16, 2.17)	1.33 (0.01, 3.33)	0.42 (0.09, 0.62)	0.54 (0.005, 0.78)	3.14 (1.25, 9.33)	10.24 (-118.57, 99.69)
Cold spell						
P10_2d	-0.12 (-0.36, 0.09)	-0.39 (-0.82, -0.03)	-0.10 (-0.30, 0.07)	-0.36 (-0.77, -0.03)	0.65 (0.29, 1.49)	0.21 (-0.19, 0.88)
P10_3d	-0.12 (-0.41, 0.13)	-0.58 (-1.15, -0.11)	-0.10 (-0.35, 0.11)	-0.53 (-1.12, -0.10)	0.63 (0.21, 2.36)	0.14 (-0.15, 0.63)
P10_4d	-0.17 (-0.52, 0.12)	-0.49 (-1.19, 0.06)	-0.15 (-0.46, 0.10)	-0.44 (-1.16, 0.05)	0.51 (0.12, 1.83)	0.20 (-0.12, 1.40)

Definition	> 80	≤ 80	> 80	≤ 80	> 80	≤ 80
	RERI (95% CI)	RERI (95% CI)	AP (95% CI)	AP (95% CI)	S (95% CI)	S (95% CI)
P7.5_2d	-0.11 (-0.35, 0.14)	-0.47 (-0.99, -0.02)	-0.09 (-0.30, 0.11)	-0.43 (-0.97, -0.01)	0.67 (0.24, 2.18)	0.17 (-0.17, 0.89)
P7.5_3d	-0.25 (-0.62, 0.06)	-0.87 (-1.76, -0.26)	-0.22 (-0.57, 0.05)	-0.85 (-1.86, -0.22)	0.38 (0.04, 1.33)	0.03 (-0.20, 0.43)
P7.5_4d	-0.38 (-0.86, 0.07)	-1.40 (-2.71, -0.48)	-0.33 (-0.81, 0.05)	-1.43 (-3.04, -0.43)	0.26 (-0.05, 1.42)	-0.01 (-0.16, 0.22)
P5_2d	-0.17 (-0.51, 0.15)	-0.96 (-1.71, -0.34)	-0.15 (-0.47, 0.12)	-1.02 (-2.01, -0.31)	0.47 (-0.03, 2.56)	-0.06 (-0.32, 0.24)
P5_3d	-0.31 (-0.83, 0.16)	-1.99 (-3.60, -0.86)	-0.27 (-0.81, 0.13)	-2.45 (-5.34, -0.89)	0.28 (-0.14, 2.17)	-0.10 (-0.26, 0.03)
P5_4d	-0.52 (-1.33, 0.05)	-2.80 (-5.75, -1.00)	-0.46 (-1.35, 0.04)	-3.59 (-9.01, -1.14)	0.20 (-0.08, 1.13)	-0.09 (-0.20, 0.02)
P2.5_2d	-0.12 (-0.65, 0.32)	-0.97 (-2.08, -0.21)	-0.09 (-0.56, 0.22)	-1.11 (-2.77, -0.23)	0.72 (0.14, 3.04)	-0.16 (-0.65, 0.29)
P2.5_3d	0.18 (-0.54, 0.83)	-1.46 (-3.66, -0.23)	0.12 (-0.43, 0.47)	-1.81 (-5.58, -0.23)	1.57 (-20.54, 22.02)	-0.15 (-0.77, 0.21)
P2.5_4d	0.24 (-0.97, 1.09)	-2.42 (-8.38, -0.07)	0.15 (-0.74, 0.60)	-3.26 (-14.31, -0.08)	1.74 (-20.06, 22.19)	-0.12 (-0.84, 0.28)

[a]$PM_{2.5}$, fine particulate matter; ETEs, extreme temperature events; CI, confidence interval; RERI, relative excess risk due to interaction; AP, attributable proportion due to interaction; S, synergy index. [b]The stratification of $PM_{2.5}$ is based on a threshold value of 40.8 μg/m^3. [c]RERI and AP greater than 0, and S greater than 1 indicate a positive interaction (synergistic effect), while RERI and AP, and S smaller than 1 indicate a negative interaction.

Table S12. Sensitivity analyses: odds ratio (95% CI) of mortality from pneumonia associated with exposure to heat wave, cold spell, and $PM_{2.5}$ with O_3 adjusted[a,b]

Definition	Heat wave	Cold spell	$PM_{2.5}$[c]
	OR (95% CI)	OR (95% CI)	OR (95% CI)
P90/10_2d	1.21 (1.12, 1.29)	1.10 (1.04, 1.17)	1.013 (1.005, 1.021)
P90/10_3d	1.20 (1.11, 1.29)	1.10 (1.03, 1.17)	1.012 (1.005, 1.020)
P90/10_4d	1.20 (1.12, 1.30)	1.10 (1.03, 1.18)	1.012 (1.004, 1.020)
P92.5/7.5_2d	1.23 (1.14, 1.33)	1.10 (1.03, 1.18)	1.013 (1.005, 1.021)
P92.5/7.5_3d	1.22 (1.13, 1.32)	1.09 (1.02, 1.18)	1.012 (1.005, 1.020)
P92.5/7.5_4d	1.22 (1.12, 1.32)	1.10 (1.02, 1.20)	1.011 (1.004, 1.019)
P95/5_2d	1.32 (1.21, 1.44)	1.08 (0.996, 1.16)	1.012 (1.005, 1.020)
P95/5_3d	1.31 (1.19, 1.44)	1.08 (0.99, 1.17)	1.012 (1.004, 1.019)
P95/5_4d	1.30 (1.18, 1.43)	1.10 (0.996, 1.21)	1.011 (1.004, 1.019)
P97.5/2.5_2d	1.47 (1.30, 1.65)	1.16 (1.04, 1.28)	1.012 (1.005, 1.020)
P97.5/2.5_3d	1.53 (1.34, 1.74)	1.17 (1.03, 1.33)	1.012 (1.004, 1.020)
P97.5/2.5_4d	1.53 (1.32, 1.77)	1.19 (1.02, 1.39)	1.011 (1.003, 1.019)

[a]$PM_{2.5}$, fine particulate matter; OR, odds ratio; CI, confidence interval. [b]Lag structure up to 6 days for both heat wave and cold spell. [c]Cumulative association of $PM_{2.5}$ (lag 0-6 day) exposure with pneumonia deaths were estimated by adjusting heat wave (P90_2d) and cold spell (P10_2d).

Table S13. Sensitivity analyses: relative excess risk due to interaction of exposure to ETEs and high-level $PM_{2.5}$ on mortality from pneumonia with O_3 adjusted[a,b,c]

ETEs	RERI (95% CI)	AP (95% CI)	S (95% CI)
Heat wave			
P90_2d	0.28 (-0.04, 0.62)	0.18 (-0.03, 0.34)	1.97 (0.89, 3.86)
P90_3d	0.42 (0.07, 0.84)	0.25 (0.05, 0.41)	2.71 (1.23, 5.64)
P90_4d	0.42 (0.08, 0.85)	0.25 (0.06, 0.42)	2.74 (1.26, 6.14)
P92.5_2d	0.46 (0.11, 0.89)	0.26 (0.07, 0.42)	2.65 (1.31, 5.34)
P92.5_3d	0.57 (0.18, 1.02)	0.31 (0.12, 0.46)	3.38 (1.58, 7.38)
P92.5_4d	0.68 (0.24, 1.17)	0.36 (0.16, 0.50)	4.22 (1.92, 10.40)
P95_2d	0.49 (0.04, 0.99)	0.27 (0.03, 0.43)	2.39 (1.09, 4.63)
P95_3d	0.61 (0.16, 1.14)	0.32 (0.11, 0.48)	3.02 (1.45, 6.87)
P95_4d	0.76 (0.32, 1.32)	0.38 (0.20, 0.53)	4.06 (1.96, 11.30)
P97.5_2d	0.54 (0.02, 1.10)	0.27 (0.01, 0.45)	2.23 (1.03, 4.64)
P97.5_3d	0.72 (0.13, 1.38)	0.33 (0.07, 0.51)	2.64 (1.23, 6.29)
P97.5_4d	1.02 (0.29, 1.90)	0.43 (0.17, 0.61)	3.88 (1.59, 13.60)
Cold spell			
P10_2d	-0.18 (-0.38, -0.01)	-0.15 (-0.32, -0.01)	0.49 (0.21, 0.97)
P10_3d	-0.23 (-0.47, -0.01)	-0.20 (-0.42, -0.01)	0.40 (0.14, 0.95)
P10_4d	-0.26 (-0.56, 0.001)	-0.22 (-0.51, 0.001)	0.36 (0.09, 1.01)
P7.5_2d	-0.19 (-0.42, 0.03)	-0.16 (-0.37, 0.03)	0.46 (0.17, 1.19)
P7.5_3d	-0.39 (-0.72, -0.10)	-0.36 (-0.70, -0.08)	0.20 (-0.02, 0.63)
P7.5_4d	-0.62 (-1.05, -0.22)	-0.58 (-1.05, -0.20)	0.10 (-0.07, 0.41)
P5_2d	-0.37 (-0.66, -0.09)	-0.34 (-0.65, -0.08)	0.17 (-0.09, 0.63)
P5_3d	-0.69 (-1.22, -0.27)	-0.68 (-1.31, -0.25)	0.01 (-0.16, 0.31)
P5_4d	-0.99 (-1.82, -0.41)	-0.99 (-2.00, -0.37)	0.00 (-0.14, 0.24)
P2.5_2d	-0.37 (-0.84, 0.04)	-0.32 (-0.81, 0.03)	0.29 (-0.05, 1.19)
P2.5_3d	-0.26 (-0.99, 0.33)	-0.21 (-0.89, 0.23)	0.47 (0.02, 3.89)
P2.5_4d	-0.43 (-1.65, 0.38)	-0.34 (-1.59, 0.28)	0.36 (-0.10, 3.02)

[a]ETEs, extreme temperature events; $PM_{2.5}$, fine particulate matter; CI, confidence interval; RERI, relative excess risk due to interaction; AP, attributable proportion due to interaction; S, synergy index. [b]The stratification of $PM_{2.5}$ is based on a threshold value of 40.8 $\mu g/m^3$. [c]RERI and AP greater than 0, and S greater than 1 indicate a positive interaction (synergistic effect), while RERI and AP, and S smaller than 1 indicate a negative interaction.

Table S14. Sensitivity analyses: odds ratio (95% CI) of mortality from pneumonia associated with exposure to heat wave, cold spell, and $PM_{2.5}$ in different season[a,b,c]

Definition	$PM_{2.5}$[d]	Definition	$PM_{2.5}$[d]	*P* value[e]
	OR (95% CI)		OR (95% CI)	
P90_2d	1.039 (1.019, 1.059)	P10_2d	1.011 (1.003, 1.019)	0.01
P90_3d	1.038 (1.018, 1.058)	P10_3d	1.010 (1.002, 1.019)	0.01
P90_4d	1.037 (1.017, 1.057)	P10_4d	1.010 (1.002, 1.018)	0.02
P92.5_2d	1.036 (1.016, 1.056)	P7.5_2d	1.011 (1.003, 1.019)	0.02
P92.5_3d	1.034 (1.014, 1.054)	P7.5_3d	1.010 (1.002, 1.018)	0.03
P92.5_4d	1.033 (1.013, 1.054)	P7.5_4d	1.010 (1.001, 1.018)	0.03
P95_2d	1.031 (1.012, 1.052)	P5_2d	1.010 (1.002, 1.019)	0.06
P95_3d	1.030 (1.010, 1.051)	P5_3d	1.010 (1.002, 1.018)	0.06
P95_4d	1.030 (1.011, 1.051)	P5_4d	1.009 (1.001, 1.017)	0.05
P97.5_2d	1.029 (1.009, 1.049)	P2.5_2d	1.011 (1.002, 1.019)	0.10
P97.5_3d	1.030 (1.010, 1.050)	P2.5_3d	1.010 (1.002, 1.019)	0.08
P97.5_4d	1.031 (1.011, 1.051)	P2.5_4d	1.009 (1.001, 1.017)	0.04

[a]$PM_{2.5}$, fine particulate matter; OR, odds ratio; CI, confidence interval. [b]Lag structure up to 6 days for both heat wave and cold spell. [c]Warm season: May to October; cold: season November to March. [d]Cumulative association of $PM_{2.5}$ (lag 0-6 day) exposure with pneumonia deaths were estimated by adjusting heat wave (P90_2d) or cold spell (P10_2d). [e]Difference of odds ratio of mortality from pneumonia associated with $PM_{2.5}$ exposure between different season were estimated by the 2-sample *z* test.

Table S15. Sensitivity analyses: odds ratio (95% CI) of mortality from pneumonia associated with exposure to heat wave, cold spell, and $PM_{2.5}$ with 37.5 μg/m^3 as threshold value[a]

ETEs	$PM_{2.5}$	ETEs	Co-exposure
	OR_{01} (95% CI)	OR_{10} (95% CI)	OR_{11} (95% CI)
Heat wave			
P90_2d	1.15 (1.09, 1.22)	1.17 (1.08, 1.27)	1.65 (1.40, 1.93)
P90_3d	1.14 (1.08, 1.20)	1.13 (1.04, 1.23)	1.73 (1.47, 2.04)
P90_4d	1.14 (1.08, 1.20)	1.13 (1.04, 1.23)	1.77 (1.49, 2.09)
P92.5_2d	1.14 (1.08, 1.20)	1.16 (1.06, 1.27)	1.80 (1.52, 2.13)
P92.5_3d	1.14 (1.08, 1.20)	1.12 (1.02, 1.23)	1.89 (1.59, 2.25)
P92.5_4d	1.13 (1.07, 1.19)	1.09 (0.99, 1.21)	1.98 (1.66, 2.38)
P95_2d	1.13 (1.08, 1.19)	1.24 (1.12, 1.37)	1.89 (1.57, 2.27)
P95_3d	1.13 (1.07, 1.19)	1.18 (1.05, 1.32)	2.01 (1.66, 2.43)
P95_4d	1.12 (1.07, 1.18)	1.12 (0.99, 1.26)	2.16 (1.77, 2.63)
P97.5_2d	1.13 (1.07, 1.19)	1.33 (1.14, 1.54)	2.08 (1.69, 2.56)
P97.5_3d	1.12 (1.07, 1.18)	1.31 (1.11, 1.56)	2.34 (1.87, 2.93)
P97.5_4d	1.12 (1.06, 1.17)	1.22 (0.997, 1.49)	2.58 (2.00, 3.33)
Cold spell			
P10_2d	1.15 (1.09, 1.22)	1.25 (1.08, 1.44)	1.23 (1.13, 1.33)
P10_3d	1.14 (1.08, 1.20)	1.28 (1.08, 1.52)	1.21 (1.11, 1.32)
P10_4d	1.14 (1.08, 1.20)	1.35 (1.10, 1.64)	1.19 (1.08, 1.31)
P7.5_2d	1.14 (1.08, 1.20)	1.26 (1.07, 1.48)	1.21 (1.10, 1.33)
P7.5_3d	1.14 (1.08, 1.20)	1.46 (1.18, 1.81)	1.15 (1.04, 1.28)
P7.5_4d	1.13 (1.07, 1.19)	1.75 (1.34, 2.28)	1.11 (0.99, 1.25)
P5_2d	1.13 (1.08, 1.19)	1.32 (1.09, 1.61)	1.14 (1.02, 1.27)
P5_3d	1.13 (1.07, 1.19)	1.69 (1.28, 2.23)	1.07 (0.94, 1.22)
P5_4d	1.12 (1.07, 1.18)	2.10 (1.45, 3.05)	1.06 (0.92, 1.22)
P2.5_2d	1.13 (1.07, 1.19)	1.41 (1.10, 1.82)	1.22 (1.05, 1.42)
P2.5_3d	1.12 (1.07, 1.18)	1.60 (1.08, 2.35)	1.25 (1.04, 1.50)
P2.5_4d	1.12 (1.06, 1.17)	1.83 (0.97, 3.45)	1.28 (1.04, 1.57)

[a]ETEs, extreme temperature events; $PM_{2.5}$, fine particulate matter; CI, confidence interval; OR_{11}, co-exposure to ETEs and high-level $PM_{2.5}$; OR_{10}, exposure to ETEs; OR_{01}, exposure to high-level $PM_{2.5}$.

Table S16. Sensitivity analyses: relative excess risk due to interaction of exposure to ETEs and high-level $PM_{2.5}$ with 37.5 μg/m^3 as threshold value on mortality from pneumonia[a,b,c]

ETEs	RERI (95% CI)	AP (95% CI)	S (95% CI)
Heat wave			
P90_2d	0.33 (0.05, 0.64)	0.20 (0.04, 0.33)	2.03 (1.14, 3.43)
P90_3d	0.45 (0.18, 0.77)	0.26 (0.12, 0.39)	2.65 (1.52, 5.02)
P90_4d	0.50 (0.18, 0.86)	0.28 (0.12, 0.41)	2.87 (1.53, 5.36)
P92.5_2d	0.50 (0.19, 0.83)	0.28 (0.12, 0.40)	2.62 (1.50, 5.08)
P92.5_3d	0.63 (0.26, 1.00)	0.33 (0.17, 0.45)	3.44 (1.81, 6.73)
P92.5_4d	0.76 (0.38, 1.18)	0.38 (0.23, 0.50)	4.44 (2.28, 10.58)
P95_2d	0.52 (0.12, 0.95)	0.27 (0.08, 0.42)	2.40 (1.27, 4.55)
P95_3d	0.70 (0.29, 1.15)	0.35 (0.18, 0.48)	3.25 (1.74, 6.87)
P95_4d	0.92 (0.43, 1.41)	0.43 (0.25, 0.55)	4.85 (2.26, 14.44)
P97.5_2d	0.62 (0.15, 1.17)	0.30 (0.08, 0.46)	2.37 (1.23, 4.68)
P97.5_3d	0.90 (0.29, 1.55)	0.39 (0.16, 0.53)	3.06 (1.48, 6.34)
P97.5_4d	1.24 (0.57, 2.04)	0.48 (0.27, 0.63)	4.67 (2.07, 14.09)
Cold spell			
P10_2d	-0.18 (-0.40, 0.01)	-0.14 (-0.34, 0.01)	0.56 (0.29, 1.03)
P10_3d	-0.22 (-0.49, 0.04)	-0.18 (-0.42, 0.03)	0.49 (0.20, 1.16)
P10_4d	-0.29 (-0.63, 0.01)	-0.24 (-0.54, 0.01)	0.40 (0.15, 1.05)
P7.5_2d	-0.19 (-0.45, 0.04)	-0.16 (-0.39, 0.04)	0.52 (0.23, 1.23)
P7.5_3d	-0.45 (-0.81, -0.09)	-0.39 (-0.74, -0.08)	0.25 (0.05, 0.69)
P7.5_4d	-0.77 (-1.35, -0.33)	-0.69 (-1.25, -0.28)	0.13 (-0.01, 0.39)
P5_2d	-0.32 (-0.67, -0.05)	-0.28 (-0.63, -0.04)	0.31 (0.04, 0.83)
P5_3d	-0.75 (-1.33, -0.24)	-0.70 (-1.32, -0.20)	0.09 (-0.05, 0.43)
P5_4d	-1.16 (-2.16, -0.48)	-1.09 (-2.24, -0.40)	0.05 (-0.05, 0.28)
P2.5_2d	-0.32 (-0.81, 0.12)	-0.26 (-0.73, 0.09)	0.41 (0.07, 1.64)
P2.5_3d	-0.47 (-1.30, 0.18)	-0.37 (-1.15, 0.13)	0.35 (0.04, 1.87)
P2.5_4d	-0.67 (-2.45, 0.35)	-0.53 (-2.18, 0.24)	0.29 (-0.01, 2.11)

[a]$PM_{2.5}$, fine particulate matter; ETEs, extreme temperature events; CI, confidence interval; RERI, relative excess risk due to interaction; AP, attributable proportion due to interaction; S, synergy index. [b]The stratification of $PM_{2.5}$ is based on a threshold value of 37.5 μg/m^3. [c]RERI and AP greater than 0, and S greater than 1 indicate a positive interaction (synergistic effect), while RERI and AP, and S smaller than 1 indicate a negative interaction.

Table S17. Sensitivity analyses: excess fraction and number of excess deaths of pneumonia from exposure to ETEs and high-level $PM_{2.5}$ with 37.5 μg/m^3 as threshold value[a,b]

Definition	Number of excess death (N)			Excess fraction (%)		
	$PM_{2.5}$	ETEs	Co-expsure	$PM_{2.5}$	ETEs	Co-expsure
Heat wave						
P90_2d	3,107 (2,010, 4,209)	447 (189, 671)	267 (186, 339)	6.19 (4.14, 8.24)	0.89 (0.41, 1.30)	0.53 (0.36, 0.67)
P90_3d	3,013 (1,873, 4,184)	315 (116, 504)	260 (190, 326)	6.00 (3.57, 8.11)	0.63 (0.20, 1.04)	0.52 (0.37, 0.64)
P90_4d	3,008 (1,871, 4,033)	278 (85, 448)	249 (179, 307)	5.99 (3.61, 8.36)	0.55 (0.14, 0.91)	0.50 (0.36, 0.61)
P92.5_2d	3,062 (1,840, 4,305)	323 (132, 490)	269 (196, 333)	6.10 (3.91, 8.37)	0.64 (0.26, 1.00)	0.54 (0.41, 0.66)
P92.5_3d	3,032 (1,811, 4,129)	209 (40, 363)	263 (200, 320)	6.04 (3.71, 8.39)	0.42 (0.06, 0.72)	0.52 (0.40, 0.64)
P92.5_4d	2,955 (1,672, 4,121)	134 (-21, 263)	258 (198, 310)	5.89 (3.60, 8.16)	0.27 (-0.03, 0.53)	0.51 (0.39, 0.62)
P95_2d	3,017 (1,814, 4,195)	285 (152, 421)	240 (174, 296)	6.01 (3.51, 8.40)	0.57 (0.30, 0.80)	0.48 (0.35, 0.58)
P95_3d	3,002 (1,764, 4,158)	178 (71, 284)	230 (172, 278)	5.98 (3.48, 8.35)	0.36 (0.11, 0.58)	0.46 (0.34, 0.55)
P95_4d	2,866 (1,737, 4,144)	99 (-13, 192)	223 (168, 268)	5.71 (3.31, 8.14)	0.20 (-0.01, 0.39)	0.44 (0.34, 0.53)
P97.5_2d	3,035 (1,831, 4,261)	165 (81, 237)	195 (148, 237)	6.05 (3.48, 8.51)	0.33 (0.17, 0.48)	0.39 (0.29, 0.47)
P97.5_3d	2,980 (1,834, 4,124)	115 (45, 175)	186 (144, 220)	5.94 (3.52, 8.31)	0.23 (0.09, 0.35)	0.37 (0.29, 0.45)
P97.5_4d	2,868 (1,541, 4,086)	64 (5, 119)	167 (128, 200)	5.71 (3.20, 8.12)	0.13 (0.00, 0.23)	0.33 (0.26, 0.40)
Cold spell						
P10_2d	3,107 (1,955, 4,183)	320 (113, 499)	739 (438, 1,006)	6.19 (3.92, 8.36)	0.64 (0.23, 1.00)	1.47 (0.88, 2.07)
P10_3d	3,013 (1,743, 4,150)	233 (66, 378)	519 (273, 752)	6.00 (3.65, 8.17)	0.46 (0.15, 0.75)	1.03 (0.54, 1.47)
P10_4d	3,008 (1,824, 4,157)	198 (67, 318)	375 (161, 562)	5.99 (3.66, 8.18)	0.39 (0.13, 0.63)	0.75 (0.37, 1.12)

Definition	Number of excess death (N)			Excess fraction (%)		
	$PM_{2.5}$	ETEs	Co-expsure	$PM_{2.5}$	ETEs	Co-expsure
P7.5_2d	3,062 (1,869, 4,241)	252 (74, 421)	495 (260, 704)	6.10 (3.73, 8.30)	0.50 (0.17, 0.83)	0.99 (0.53, 1.41)
P7.5_3d	3,032 (1,847, 4,158)	235 (105, 352)	268 (84, 454)	6.04 (3.68, 8.33)	0.47 (0.21, 0.71)	0.53 (0.11, 0.86)
P7.5_4d	2,955 (1,749, 4,061)	221 (113, 318)	153 (-15, 307)	5.89 (3.38, 8.09)	0.44 (0.25, 0.63)	0.30 (-0.03, 0.61)
P5_2d	3,017 (1,822, 4,165)	201 (59, 345)	229 (26, 406)	6.01 (3.49, 8.42)	0.40 (0.11, 0.67)	0.46 (0.09, 0.82)
P5_3d	3,002 (1,873, 4,128)	207 (93, 301)	87 (-73, 235)	5.98 (3.66, 8.08)	0.41 (0.19, 0.60)	0.17 (-0.16, 0.46)
P5_4d	2,866 (1,590, 4,026)	178 (89, 253)	61 (-87, 194)	5.71 (3.40, 7.89)	0.35 (0.18, 0.51)	0.12 (-0.17, 0.37)
P2.5_2d	3,035 (1,691, 4,198)	132 (40, 222)	170 (41, 293)	6.05 (3.85, 8.49)	0.26 (0.07, 0.45)	0.34 (0.08, 0.57)
P2.5_3d	2,980 (1,742, 4,053)	97 (16, 167)	136 (18, 235)	5.94 (3.43, 8.35)	0.19 (0.03, 0.34)	0.27 (0.05, 0.46)
P2.5_4d	2,868 (1,640, 4,075)	55 (-5, 108)	105 (17, 187)	5.71 (3.02, 8.19)	0.11 (-0.01, 0.21)	0.21 (0.03, 0.37)

[a]$PM_{2.5}$, fine particulate matter; ETEs, extreme temperature events. [b]The stratification of $PM_{2.5}$ is based on a threshold value of 37.5 μg/m^3.

Table S18. Sensitivity analyses (using air temperature): odds ratio (95% CI) of mortality from pneumonia associated with exposure to heat wave, cold spell, and $PM_{2.5}$[a,b]

ETEs	$PM_{2.5}$	ETEs	Co-exposure
	OR_{01} (95% CI)	OR_{10} (95% CI)	OR_{11} (95% CI)
Heat wave			
P90_2d	1.13 (1.08, 1.20)	1.18 (1.09, 1.28)	1.74 (1.44, 2.10)
P90_3d	1.12 (1.07, 1.18)	1.14 (1.05, 1.24)	1.83 (1.50, 2.24)
P90_4d	1.11 (1.06, 1.17)	1.14 (1.05, 1.24)	1.87 (1.52, 2.30)
P92.5_2d	1.13 (1.07, 1.19)	1.26 (1.15, 1.37)	1.82 (1.49, 2.23)
P92.5_3d	1.12 (1.07, 1.18)	1.20 (1.10, 1.32)	1.86 (1.51, 2.30)
P92.5_4d	1.12 (1.06, 1.18)	1.22 (1.11, 1.34)	1.93 (1.56, 2.40)
P95_2d	1.13 (1.08, 1.19)	1.28 (1.16, 1.42)	1.92 (1.55, 2.39)
P95_3d	1.13 (1.07, 1.18)	1.26 (1.13, 1.40)	2.05 (1.63, 2.57)
P95_4d	1.12 (1.06, 1.17)	1.23 (1.09, 1.39)	2.07 (1.64, 2.61)
P97.5_2d	1.11 (1.06, 1.17)	1.41 (1.23, 1.63)	2.35 (1.81, 3.05)
P97.5_3d	1.11 (1.05, 1.16)	1.39 (1.18, 1.63)	2.61 (1.99, 3.42)
P97.5_4d	1.10 (1.05, 1.16)	1.30 (1.08, 1.57)	2.59 (1.95, 3.42)
Cold spell			
P10_2d	1.13 (1.08, 1.20)	1.25 (1.10, 1.41)	1.19 (1.09, 1.30)
P10_3d	1.12 (1.07, 1.18)	1.26 (1.09, 1.46)	1.17 (1.07, 1.28)
P10_4d	1.11 (1.06, 1.17)	1.26 (1.07, 1.49)	1.18 (1.07, 1.30)
P7.5_2d	1.13 (1.07, 1.19)	1.22 (1.05, 1.41)	1.17 (1.06, 1.29)
P7.5_3d	1.12 (1.07, 1.18)	1.36 (1.14, 1.63)	1.13 (1.01, 1.26)
P7.5_4d	1.12 (1.06, 1.18)	1.50 (1.20, 1.88)	1.10 (0.98, 1.24)
P5_2d	1.13 (1.08, 1.19)	1.40 (1.18, 1.66)	1.10 (0.98, 1.24)
P5_3d	1.13 (1.07, 1.18)	1.71 (1.36, 2.16)	1.01 (0.88, 1.15)
P5_4d	1.12 (1.06, 1.17)	1.99 (1.47, 2.68)	1.01 (0.87, 1.18)
P2.5_2d	1.11 (1.06, 1.17)	1.42 (1.14, 1.78)	1.14 (0.97, 1.34)
P2.5_3d	1.11 (1.05, 1.16)	1.47 (1.06, 2.03)	1.19 (0.98, 1.44)
P2.5_4d	1.10 (1.05, 1.16)	1.88 (1.15, 3.08)	1.17 (0.94, 1.46)

[a]ETEs, extreme temperature events; $PM_{2.5}$, fine particulate matter; CI, confidence interval; OR_{11}, co-exposure to ETEs and high-level $PM_{2.5}$; OR_{10}, exposure to ETEs; OR_{01}, exposure to high-level $PM_{2.5}$. [b]ETEs Definition using air temperature instead of the HI.

Table S19. Sensitivity analyses (using air temperature): relative excess risk due to interaction of exposure to ETEs and high-level $PM_{2.5}$[a,b,c,d]

ETEs	RERI (95% CI)	AP (95% CI)	S (95% CI)
Heat wave			
P90_2d	0.43 (0.09, 0.79)	0.25 (0.06, 0.39)	2.36 (1.24, 4.29)
P90_3d	0.57 (0.22, 1.01)	0.31 (0.14, 0.45)	3.17 (1.70, 6.63)
P90_4d	0.62 (0.24, 1.07)	0.33 (0.15, 0.47)	3.42 (1.78, 6.69)
P92.5_2d	0.44 (0.02, 0.87)	0.24 (0.01, 0.39)	2.14 (1.05, 3.76)
P92.5_3d	0.54 (0.15, 0.97)	0.29 (0.10, 0.43)	2.64 (1.38, 4.98)
P92.5_4d	0.59 (0.17, 1.10)	0.31 (0.11, 0.47)	2.75 (1.42, 5.49)
P95_2d	0.51 (0.06, 1.00)	0.27 (0.04, 0.43)	2.23 (1.13, 4.17)
P95_3d	0.66 (0.16, 1.19)	0.32 (0.10, 0.47)	2.71 (1.37, 5.12)
P95_4d	0.71 (0.19, 1.27)	0.35 (0.11, 0.50)	3.02 (1.41, 6.13)
P97.5_2d	0.83 (0.21, 1.58)	0.35 (0.11, 0.52)	2.57 (1.33, 4.89)
P97.5_3d	1.12 (0.38, 1.96)	0.43 (0.18, 0.59)	3.26 (1.58, 6.90)
P97.5_4d	1.18 (0.43, 2.08)	0.46 (0.21, 0.61)	3.93 (1.77, 10.72)
Cold spell			
P10_2d	-0.19 (-0.37, -0.005)	-0.16 (-0.32, -0.004)	0.50 (0.26, 0.98)
P10_3d	-0.21 (-0.46, -0.01)	-0.18 (-0.41, -0.01)	0.45 (0.17, 0.96)
P10_4d	-0.19 (-0.48, 0.06)	-0.16 (-0.42, 0.05)	0.49 (0.17, 1.45)
P7.5_2d	-0.17 (-0.41, 0.02)	-0.15 (-0.35, 0.02)	0.50 (0.19, 1.12)
P7.5_3d	-0.36 (-0.67, -0.07)	-0.32 (-0.65, -0.05)	0.26 (0.01, 0.77)
P7.5_4d	-0.52 (-0.96, -0.14)	-0.47 (-0.92, -0.12)	0.17 (-0.04, 0.59)
P5_2d	-0.42 (-0.75, -0.12)	-0.38 (-0.71, -0.10)	0.20 (-0.03, 0.59)
P5_3d	-0.83 (-1.35, -0.38)	-0.83 (-1.45, -0.35)	0.01 (-0.13, 0.25)
P5_4d	-1.09 (-1.88, -0.44)	-1.08 (-2.06, -0.40)	0.01 (-0.10, 0.24)
P2.5_2d	-0.40 (-0.84, -0.03)	-0.35 (-0.81, -0.02)	0.26 (-0.07, 0.92)
P2.5_3d	-0.38 (-1.02, 0.19)	-0.32 (-0.95, 0.14)	0.33 (-0.03, 2.16)
P2.5_4d	-0.81 (-2.19, 0.09)	-0.69 (-2.18, 0.07)	0.18 (-0.05, 1.27)

[a]$PM_{2.5}$, fine particulate matter; ETEs, extreme temperature events; CI, confidence interval; RERI, relative excess risk due to interaction; AP, attributable proportion due to interaction; S, synergy index. [b]The stratification of $PM_{2.5}$ is based on a threshold value of 40.8 μg/m^3. [c]RERI and AP greater than 0, and S greater than 1 indicate a positive interaction (synergistic effect), while RERI and AP, and S smaller than 1 indicate a negative interaction. [d]ETEs definitions using air temperature instead of the HI.

Table S20. Sensitivity analyses (using apparent temperature): odds ratio (95% CI) of mortality from pneumonia associated with exposure to heat wave, cold spell, and $PM_{2.5}$[a,b]

ETEs	$PM_{2.5}$	ETEs	Co-exposure
	OR_{01} (95% CI)	OR_{10} (95% CI)	OR_{11} (95% CI)
Heat wave			
P90_2d	1.13 (1.07, 1.20)	1.19 (1.09, 1.28)	1.66 (1.37, 2.01)
P90_3d	1.12 (1.06, 1.18)	1.15 (1.06, 1.25)	1.76 (1.44, 2.14)
P90_4d	1.12 (1.06, 1.17)	1.15 (1.05, 1.25)	1.82 (1.48, 2.22)
P92.5_2d	1.13 (1.07, 1.19)	1.19 (1.09, 1.30)	1.86 (1.52, 2.27)
P92.5_3d	1.12 (1.07, 1.18)	1.16 (1.06, 1.27)	1.90 (1.54, 2.33)
P92.5_4d	1.12 (1.06, 1.17)	1.16 (1.05, 1.28)	1.96 (1.59, 2.42)
P95_2d	1.12 (1.07, 1.18)	1.25 (1.13, 1.38)	1.98 (1.60, 2.45)
P95_3d	1.12 (1.06, 1.18)	1.20 (1.08, 1.34)	2.10 (1.68, 2.63)
P95_4d	1.11 (1.06, 1.17)	1.17 (1.04, 1.32)	2.24 (1.78, 2.83)
P97.5_2d	1.11 (1.06, 1.17)	1.38 (1.20, 1.59)	2.12 (1.66, 2.70)
P97.5_3d	1.11 (1.06, 1.17)	1.40 (1.19, 1.64)	2.28 (1.77, 2.94)
P97.5_4d	1.10 (1.05, 1.16)	1.34 (1.11, 1.61)	2.42 (1.86, 3.17)
Cold spell			
P10_2d	1.13 (1.07, 1.20)	1.23 (1.08, 1.40)	1.22 (1.12, 1.33)
P10_3d	1.12 (1.06, 1.18)	1.26 (1.08, 1.46)	1.19 (1.09, 1.31)
P10_4d	1.12 (1.06, 1.17)	1.30 (1.09, 1.55)	1.18 (1.07, 1.31)
P7.5_2d	1.13 (1.07, 1.19)	1.26 (1.10, 1.45)	1.18 (1.07, 1.30)
P7.5_3d	1.12 (1.07, 1.18)	1.38 (1.16, 1.65)	1.12 (1.01, 1.25)
P7.5_4d	1.12 (1.06, 1.17)	1.60 (1.27, 2.01)	1.08 (0.96, 1.22)
P5_2d	1.12 (1.07, 1.18)	1.33 (1.12, 1.57)	1.08 (0.96, 1.22)
P5_3d	1.12 (1.06, 1.18)	1.60 (1.27, 2.02)	1.003 (0.87, 1.15)
P5_4d	1.11 (1.06, 1.17)	1.99 (1.47, 2.69)	0.96 (0.82, 1.13)
P2.5_2d	1.11 (1.06, 1.17)	1.41 (1.13, 1.76)	1.19 (0.998, 1.41)
P2.5_3d	1.11 (1.06, 1.17)	1.71 (1.21, 2.41)	1.21 (0.98, 1.48)
P2.5_4d	1.10 (1.05, 1.16)	2.14 (1.23, 3.71)	1.22 (0.98, 1.54)

[a]ETEs, extreme temperature events; $PM_{2.5}$, fine particulate matter; CI, confidence interval; OR_{11}, co-exposure to ETEs and high-level $PM_{2.5}$; OR_{10}, exposure to ETEs; OR_{01}, exposure to high-level $PM_{2.5}$. [b]ETEs Definition using apparent temperature instead of the HI.

Table S21. Sensitivity analyses (using apparent temperature): relative excess risk due to interaction of exposure to ETEs and high-level $PM_{2.5}$[a,b,c,d]

ETEs	RERI (95% CI)	AP (95% CI)	S (95% CI)
Heat wave			
P90_2d	0.34 (0.02, 0.71)	0.21 (0.01, 0.36)	2.07 (1.06, 3.78)
P90_3d	0.49 (0.16, 0.87)	0.28 (0.11, 0.41)	2.82 (1.51, 5.45)
P90_4d	0.55 (0.19, 0.99)	0.30 (0.12, 0.45)	3.09 (1.59, 6.37)
P92.5_2d	0.55 (0.16, 0.98)	0.29 (0.11, 0.44)	2.73 (1.43, 5.05)
P92.5_3d	0.61 (0.22, 1.06)	0.32 (0.14, 0.46)	3.16 (1.63, 6.25)
P92.5_4d	0.68 (0.26, 1.12)	0.35 (0.16, 0.48)	3.45 (1.69, 6.96)
P95_2d	0.61 (0.18, 1.09)	0.31 (0.11, 0.46)	2.64 (1.40, 4.78)
P95_3d	0.78 (0.31, 1.29)	0.37 (0.18, 0.51)	3.41 (1.78, 6.78)
P95_4d	0.96 (0.45, 1.57)	0.43 (0.25, 0.56)	4.37 (2.21, 9.81)
P97.5_2d	0.63 (0.03, 1.26)	0.30 (0.02, 0.48)	2.27 (1.06, 4.40)
P97.5_3d	0.77 (0.19, 1.53)	0.34 (0.10, 0.52)	2.50 (1.28, 5.38)
P97.5_4d	0.98 (0.33, 1.74)	0.40 (0.18, 0.57)	3.22 (1.56, 7.76)
Cold spell			
P10_2d	-0.15 (-0.36, 0.04)	-0.12 (-0.30, 0.03)	0.60 (0.31, 1.17)
P10_3d	-0.19 (-0.44, 0.04)	-0.16 (-0.38, 0.03)	0.50 (0.21, 1.20)
P10_4d	-0.24 (-0.52, 0.04)	-0.20 (-0.46, 0.03)	0.44 (0.13, 1.15)
P7.5_2d	-0.21 (-0.42, -0.002)	-0.18 (-0.38, -0.002)	0.46 (0.18, 0.99)
P7.5_3d	-0.38 (-0.72, -0.12)	-0.34 (-0.68, -0.10)	0.25 (0.00, 0.63)
P7.5_4d	-0.64 (-1.07, -0.25)	-0.59 (-1.06, -0.22)	0.11 (-0.06, 0.39)
P5_2d	-0.37 (-0.68, -0.08)	-0.34 (-0.67, -0.07)	0.18 (-0.09, 0.65)
P5_3d	-0.72 (-1.21, -0.32)	-0.72 (-1.31, -0.30)	0.00 (-0.16, 0.28)
P5_4d	-1.14 (-1.94, -0.53)	-1.18 (-2.26, -0.50)	-0.03 (-0.15, 0.15)
P2.5_2d	-0.34 (-0.84, 0.11)	-0.28 (-0.78, 0.09)	0.36 (0.00, 1.56)
P2.5_3d	-0.62 (-1.42, 0.07)	-0.51 (-1.37, 0.05)	0.25 (-0.03, 1.22)
P2.5_4d	-1.01 (-2.62, 0.12)	-0.83 (-2.46, 0.08)	0.18 (-0.02, 1.19)

[a]$PM_{2.5}$, fine particulate matter; ETEs, extreme temperature events; CI, confidence interval; RERI, relative excess risk due to interaction; AP, attributable proportion due to interaction; S, synergy index. [b]The stratification of $PM_{2.5}$ is based on a threshold value of 40.8 μg/m^3. [c]RERI and AP greater than 0, and S greater than 1 indicate a positive interaction (synergistic effect), while RERI and AP, and S smaller than 1 indicate a negative interaction. [d]ETEs definitions using apparent temperature instead of the HI.

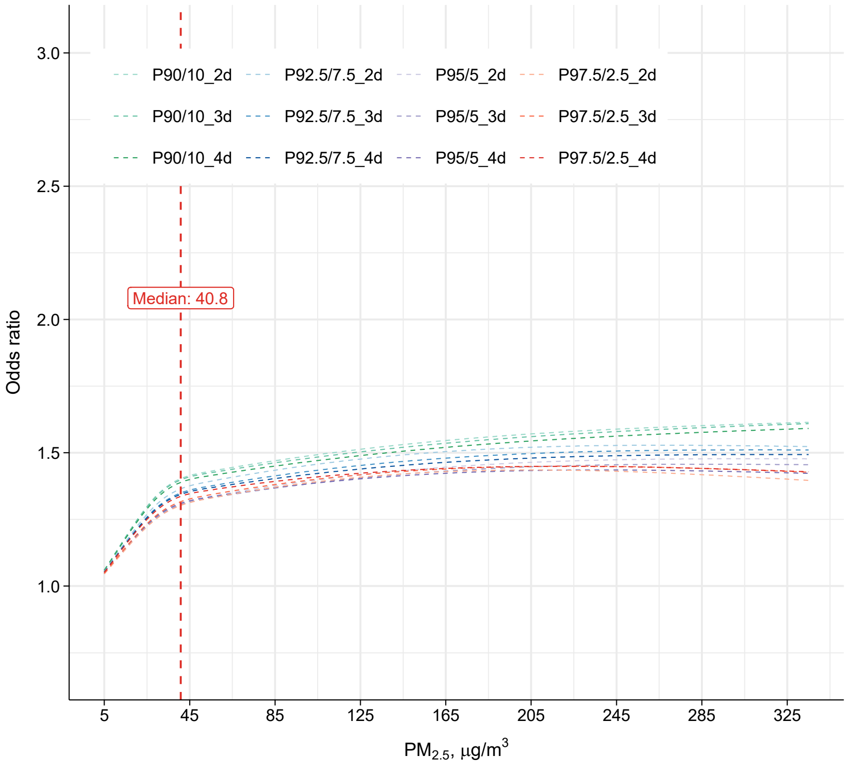

Figure S1. Exposure-response curve of the association between exposure to $PM_{2.5}$ and pneumonia mortality. The dashed line represents the OR of pneumonia mortality associated with exposure to $PM_{2.5}$ with adjustment for extreme temperature events. The shaded region represents the 95% CI with adjustment for heat wave (P95_3d) and cold spell (P5_3d). The horizontal black line represents the OR of 1. $PM_{2.5}$, fine particulate matter.

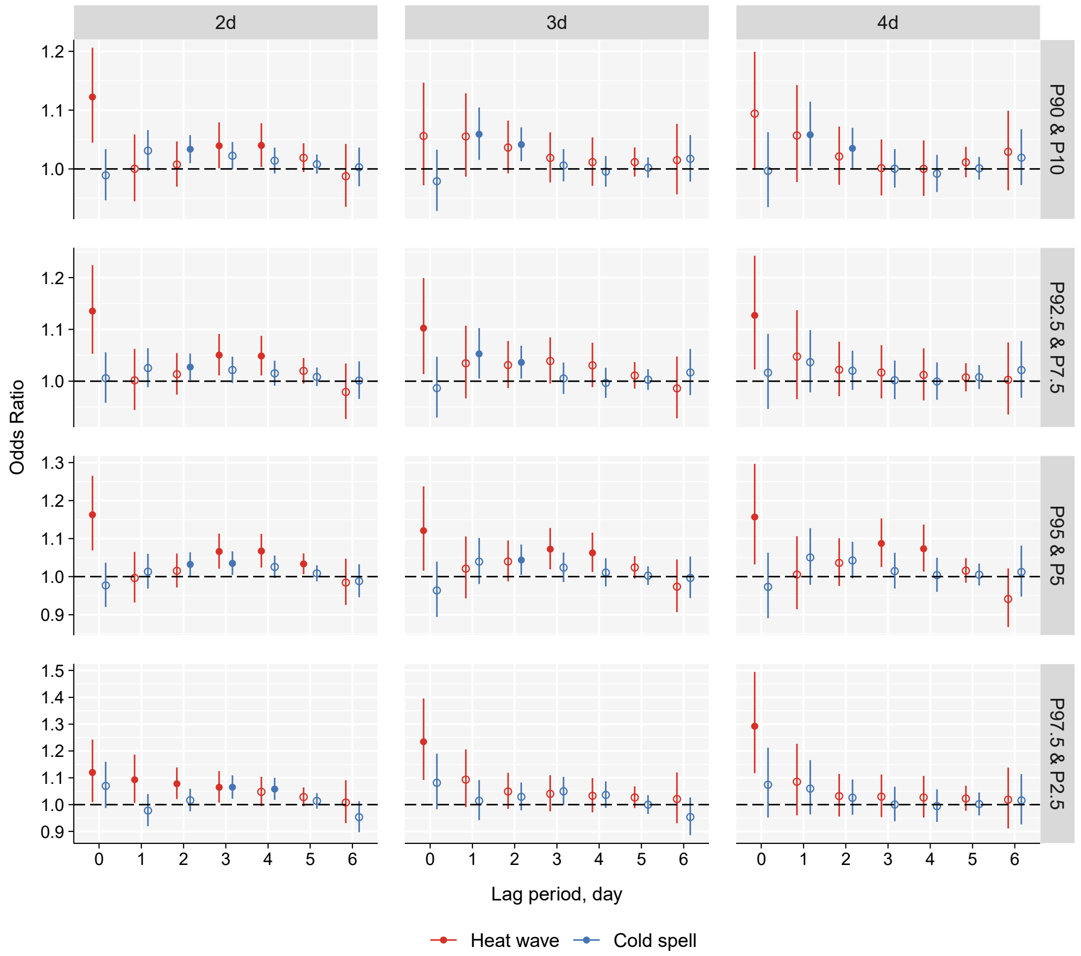

Figure S2. Overall lag structure for the association of ETE exposure with pneumonia mortality in Jiangsu province, China, during 2015-2022. Solid markers indicate statistical significance, while hollow markers represent non-significance.

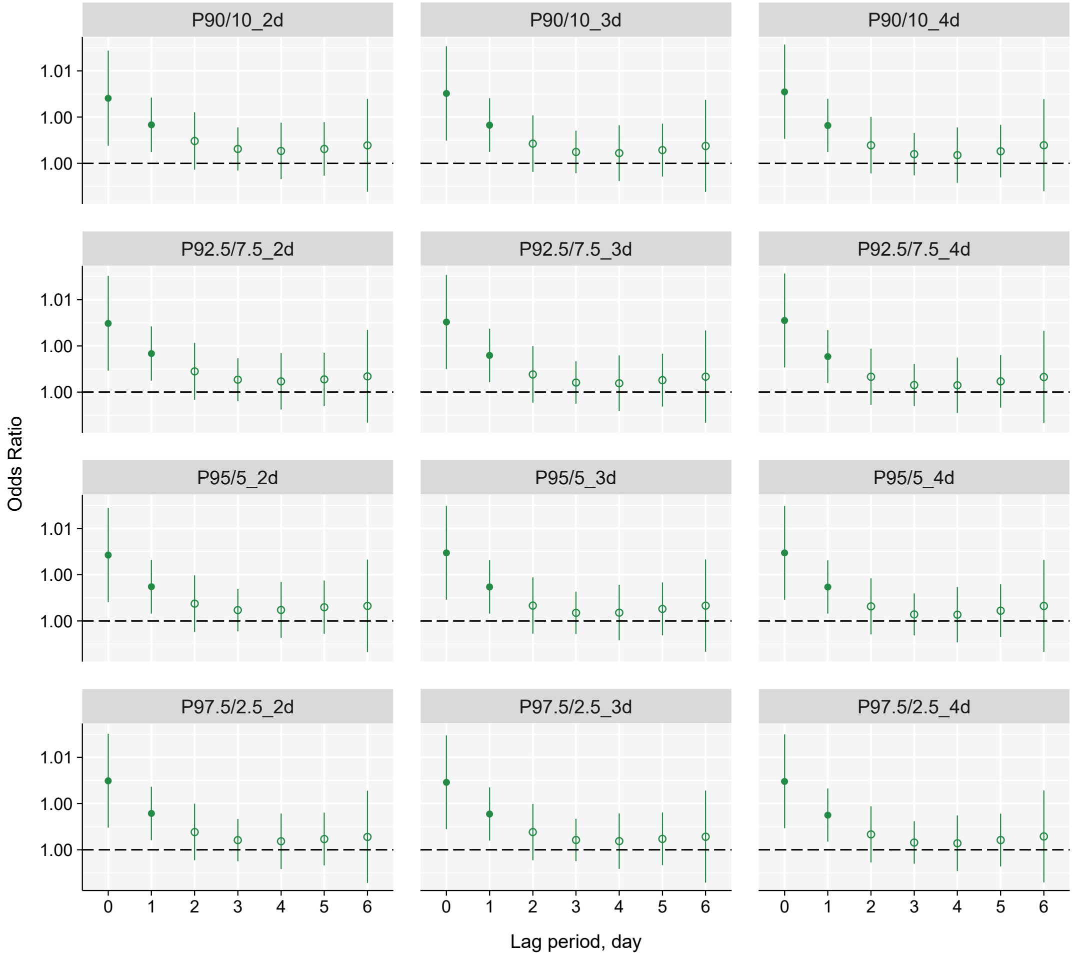

Figure S3. Overall lag structure for the association of $PM_{2.5}$ exposure with pneumonia mortality in Jiangsu province, China, during 2015-2022. Solid markers indicate statistical significance, while hollow markers represent non-significance. Associations were estimated as odds ratio and 95% confidence intervals per 10 μg/m^3 increase in $PM_{2.5}$. $PM_{2.5}$, fine particulate matter.
